# Assessment of Nutrient Intake and Diet Quality in Adolescent Dietary Supplement Users vs. Non-Users: The CRO-PALS Longitudinal Study

**DOI:** 10.3390/nu15122783

**Published:** 2023-06-17

**Authors:** Željana Mudnić, Amanda Gaši, Josip Rešetar, Jasenka Gajdoš Kljusurić, Marjeta Mišigoj-Duraković, Maroje Sorić, Ines Panjkota Krbavčić, Zvonimir Šatalić

**Affiliations:** 1 Faculty of Food Technology and Biotechnology, University of Zagreb, Pierottijeva 6, 10000 Zagreb, Croatia; 2 Faculty of Pharmacy and Biochemistry, University of Zagreb, Ante Kovačića 1, 10000 Zagreb, Croatia; 3 Faculty of Kinesiology, University of Zagreb, Horvaćanski zavoj 15, 10000 Zagreb, Croatia; 4 Faculty of Sport, University of Ljubljana, Gortanova ulica 22, 1000 Ljubljana, Slovenia

**Keywords:** dietary supplements, adolescence, diet quality, fast food, fruits and vegetables, sweetened drink, anthropometry

## Abstract

Dietary supplement users tend to have a better diet quality and overall prudent lifestyle. The main goals of this research were to report the prevalence and type of dietary supplements among Croatian adolescents and to examine the differences in the diet quality among dietary supplement users vs. non-users at the beginning (15/16 y) and at the end of high school education (18/19 y). This research is based on results of the longitudinal CRO-PALS study in which 607 adolescents participated, who had complete dietary, anthropometric, and physical activity data at the beginning (15/16 y) and at the end of their high school education (18/19 y). The dietary assessment method used was a single multi-pass 24 h recall. Dietary supplement users were divided into two groups for the purposes of statistical analysis—users of vitamin and multivitamin preparations (VMV) and users of mineral and multivitamin preparations (MMV). As they aged, there was an increase in the consumption of dietary supplements, and the most frequently used preparation in both age groups was vitamin C (23.7% of users). Dietary supplement users had a higher intake of non-carbonated sweetened drinks and a lower intake of fruits and vegetables in both genders and both age groups. Fast food intake was higher among dietary supplement girl users and boys who were not dietary supplements users in both age groups. Dietary supplement users had a higher achieved average intake of most micronutrients (values obtained only from food) in both genders and both age groups (with exceptions for certain vitamins and minerals). By observing other parameters for assessing the diet quality in this research, we can conclude that girls who do not use dietary supplements have a better diet quality in both age groups.

## 1. Introduction

According to the definition of the European Food Safety Authority (EFSA), dietary supplements are preparations that contain a concentrated source of nutrients, primarily vitamins and minerals, but may also contain other substances that have a physiological and/or nutritional effect on the human body [1]. Dietary supplements are intended for the individual as a supplement in nutritional deficiency but also for maintaining an adequate intake of certain nutrients, and they serve as support for diseases and/or specific conditions [1]. Today, there is a large number of different dietary supplements on the market, which can cause confusion in identifying the real need, proper use, and selection of dietary supplements [2,3]. The consumption of dietary supplements has become increasingly popular in developed countries, and vitamin–mineral preparations are the most widely used. Mazurek et al. [4] state that almost half of the adult population in the USA and more than 30% of children and adolescents use dietary supplements. In the same research state, that in Europe, the share of users in the adult population is between 2% and 66%, while the share of users in children and adolescents is between 16% and 45%. Most of the studies have followed the consumption of dietary supplements in the adult population, while data for adolescents are lacking and were mainly conducted in the USA [4]. Regarding dietary supplement use in Croatia, there is a study conducted by Pavičić et al. [5]. This descriptive, cross-sectional research was conducted with students of the medical and non-medical sciences in Rijeka in 2017. The prevalence of the use of dietary supplements among the respondents was 30.5%, which turned out to be lower compared to the prevalence of the use of dietary supplements among Serbian (68.1%), Australian (56%) and American (52%) students, but higher compared to Portuguese students (16%). The most commonly used dietary supplements reported by Pavičić et al. were vitamins [5].

Among the dietary supplement users, different motives for consumption have been stated. Research on the use of dietary supplements at the Europe and USA level has shown that the most common motive for consumption is the improvement and maintenance of overall health. In addition, respondents mention the strengthening of immunity, as a supplement to nutrition, the prevention of health problems, the improvement of physical and mental ability, and for relaxation, stress, and sleep [6,7,8]. In addition, research shows that use is more common in women than in men, and in adults with a higher level of education. Additionally, people who consume dietary supplements are more likely to engage in physical activity, have better eating habits and lower body mass index values, and are less likely to smoke, unlike dietary supplement non-users [9]. On the other hand, considering the consumption of dietary supplements in children and adolescents, Mazurek et al. state that adolescents mostly decide independently whether and which dietary supplements they will consume, which is primarily based on advertisements from various media and through the recommendation of peers. With children, it is the parents who decide whether and which supplements the child will consume [4]. Adolescence is a critical period of growth and development in which large and rapid changes occur—physical, cognitive, and social. In order to enable adolescents to reach their maximum growth potential, they need to be provided with enough energy, as well as macro- and micronutrients. Eating patterns during adolescence, whether healthy or unhealthy, tend to track into adulthood. During adolescence, there are changes in the eating habits of adolescents, and they begin to differ from the eating habits of childhood; for example, there is a decrease in the appetite for a sweet taste [10].

Vegetables and fruits are key components of a healthy diet, and adequate intake reduces the risk of non-communicable diseases (such as type 2 diabetes), while, on the other hand, lowers total daily energy intake, due to low energy density, increases satiety and is also associated with healthier body weight [11]. The diet of adolescents’ nowadays is high in foods with high energy density such as snacks, sweets, fast food, while, on the other hand, the consumption of plant-based food groups is low. A higher intake of foods with a high energy density entails a higher intake of fat, sodium, and added sugars, as well as a lower dietary fiber, calcium, potassium, and folate intake [10]. The Healthy Lifestyle in Europe by Nutrition in Adolescence study (HELENA study) conducted on a sample of 3000 adolescents from 10 European countries showed that adolescents consumed only half of the recommended amount of fruit and vegetable and less than two thirds of the recommended servings of dairy products (including milk), while meat (and meat products), sweets, and fatty foods were consumed above recommended restrictions [12].

Therefore, the main goals of this study were: (i) to examine the use of vitamin and multivitamin (VMV), as well as mineral and multivitamin (MMV) dietary supplements in adolescents; (ii) examine the prevalence of inadequacy of micronutrient intakes in dietary supplement users and non-users; (iii) examine changes in the intake of fruits and vegetables, fast food, non-carbonated, and carbonated sweetened beverages in dietary supplement users and non-users (in the same group of adolescents); (iv) perform an analysis of significance between micronutrient intakes, anthropometric indicators of nutritional status, the level of physical activity and intake of fruits and vegetables, fast food, non-carbonated, and carbonated sweetened beverages in adolescents (dietary supplement users and non-users) at the beginning and at the end of their high school education.

## 2. Materials and Methods

### 2.1. Subjects

The respondents presented in this study come from the longitudinal study CRO-PALS. Respondents were selected by random two-stage selection from 14 secondary schools in the area of the city of Zagreb, Croatia. The sample of respondents included 10% of all Zagreb high school students enrolled in the first grades. Two measurements were taken at the beginning and at the end of their secondary school education. Parameters such as anthropometric measurements, 24 h recall, and assessment of physical activity were collected. The total number of subjects in this study was 607. All 607 subjects had complete measurements, assessments, and 24 h recalls. The details have been described previously by Rešetar et al. [13].

### 2.2. Anthropometric Parameters

Body height, body mass, and the sum of the thickness of four skinfolds were the anthropometric parameters measured as part of the research. Body height was measured using an anthropometer (GPM, Siber-Hegner and Co., Zurich, Switzerland), and body mass was recorded using a digital scale. Body mass index is calculated as the ratio of body mass expressed in kilograms to the square of body height expressed in meters (kg/m^2^). The sum of skinfold thickness included measurements at anatomical locations: biceps, triceps, subscapular and suprailiac. All skinfolds were measured in triplicate on the right side using a Herpenden caliper (British indicators, West Sussex, UK). For the purposes of further data processing, the median of triplicates was taken from the calculation of the sum of the thickness of four skin folds. The details have been described previously by Rešetar et al. [13]. 

### 2.3. Physical Activity

The School Health Action, Planning, and Evaluation System (SHAPES) is a questionnaire that was used in the CRO-PALS longitudinal study to assess physical activity. Wong et al. described the equations for processing the results obtained through the questionnaire [14]. To determine the average daily energy expenditure of physical activity (PAEE) during the examined 7 days, the assumption of an average intensity of 4 metabolic equivalent tasks (MET) for MPA and 7 MET for VPA was taken. Given that the SHAPES questionnaire includes two items that require a 7-day recall of moderate (MPA) and vigorous intensity (VPA) of physical activity, participants had to indicate the number of 15-min steps (0–45 min) and the number of hours (from 4 to 10 h) for each day of the previous week. In this study, based on the results and the application of the equations, the average moderate-to-vigorous physical activity of each respondent during weekdays, during the entire week (weekdays including the weekend), and only during the weekend was determined. For the purposes of our work, we used the average only during workdays (not including weekend). The methods of assessing physical activity are described in more detail in a previous study by Rešetar et al. [13].

### 2.4. Assessment of Diet

The multi-pass 24 h recall was used as a dietary assessment method in this study. The method consists of a 20 min interview conducted by trained personnel (final year graduate nutrition students from the Faculty of Food and Biotechnology, University of Zagreb). During the interview, an effort was made to collect information from the respondents referring to a previous day. In our study, 24 h recalls were collected for Monday–Fridays (weekend days were intentionally omitted). During these five days, data was collected for only one day for each respondent. Moshfegh et al. described 5 standardized steps for conducting interviews [15]. When assessing the portion size, a food atlas with photos of small, medium, and large serving sizes was used [16]. A combination of national, Danish, and US food composition tables were used to determine the chemical composition of consumed food [17,18,19]. A cookbook with traditional recipes for estimation of the energy and nutrient content of composite dishes was used [20]. 

### 2.5. Reference Values

The intake of micronutrients was estimated by comparison with nutrient density limiting values (amount per 1000 kcal) of the U.S. Department of Agriculture (USDA), derived from the dietary reference intakes (DRI) for all persons older than one year. Additionally, in the Supplementary Materials, the intake of micronutrients was evaluated through comparison with EFSA referent values, using average requirement (AR) values for most micronutrients, with differences with regard to age and gender. In the absence of AR values, adequate intake (AI) values were used for vitamin B_12_, pantothenic acid, vitamins D, E and K, as well as phosphorus, magnesium, and selenium. The adequacy was evaluated by presenting % of respondents with intake below the referent value.

### 2.6. Dietary Supplements

Of all collected data for dietary supplements, this work included dietary supplements that contained the following: (i) one or more vitamins; (ii) one or more vitamins in combination with one or more minerals; (iii) energy and sports drinks, and Cedevita—as a specific fortified drink in the Republic of Croatia, which is extremely popular among the adolescent population. The chosen definition relied on the research of Sichert-Hellert et al. [21]. Before data processing, for the purposes of logistic regression, dietary supplements were divided into two groups. Group one included vitamin and multivitamin preparations (including Cedevita—water soluble powder), while group two included multivitamin-mineral preparations (including energy and sports drinks). Some respondents used several types of preparations, so they were listed as users of all registered dietary supplements (multiple times).

### 2.7. Intake of Fruits and Vegetables, Fast Food, and Sweetened Beverages

**Figure 1:**
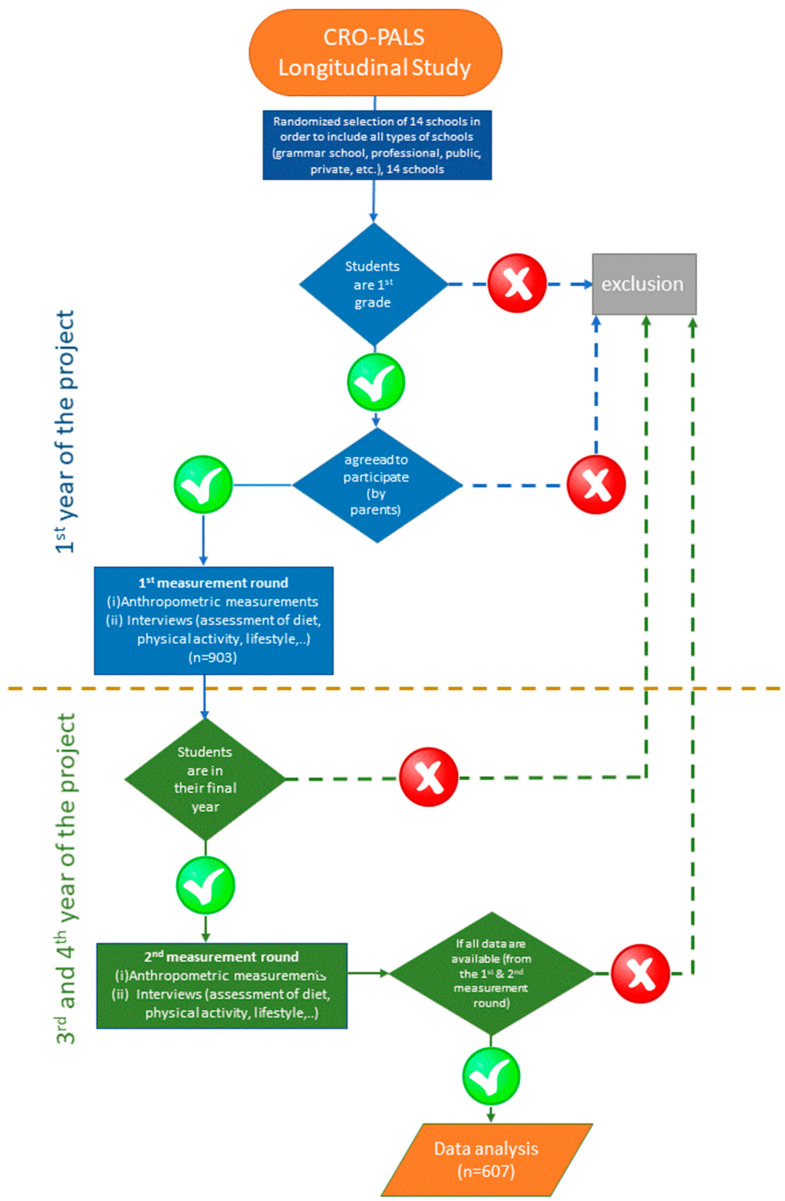
Collecting data in the longitudinal CRO-PALS study.

To assess the adolescents diet quality, data for the intake of fruits and vegetables, fast food, and sweetened drinks (carbonated and non-carbonated) were used. The total fruit intake of the respondents included fresh, cooked or canned fruit, dried fruit, nuts, and fruit juices, while the total vegetable intake included all types of vegetables (except potatoes) in fresh, cooked, or canned form. Complex dishes such as stews or smoothies were not included in the total, unless the ingredients were listed individually with quantities (in grams). The chosen definition is based on a Prynne et al. [22] study. Fruit and vegetable intake was shown in two ways: (1) g/day following the recommendations of the World Health Organization (WHO) and (2) relative to energy intake (g/1000 kcal). The total intake of fast food included various types of pizzas, sandwiches, hamburgers, salty pies, and other bakery products. In the total amount, we included dishes prepared in fast food restaurants, but also prepared at home. The exception were dishes made from whole meal bread without additional sauces such as mayonnaise or ketchup. Sweetened drinks were divided into two categories—carbonated and non-carbonated. Carbonated sweetened beverages included non-diet carbonated beverages that also contained sugar, while non-carbonated sweetened beverages included industrial fruit juices, excluding hot drinks and milk. Definitions for choosing fast food and sweetened beverages were based on previous study by Lioret et al. [23]. The intake of fast food and sweetened beverages were shown relative to energy intake (g/1000 kcal). The implementation of data collection is shown in the flow diagram that follows in Figure 1.

### 2.8. Statistical Analysis

The Microsoft Excel 2013 program with XLSTAT (2007, Statistical Software for Excel) was used for statistical processing of the data and for the logistic regression. Descriptive statistic tools were used for the basic statistical analysis of the data and included the calculation of the mean value (average), standard deviation, minimum and maximum values (ranges), and frequency. Depending on the data, to determine the significant differences (α = 0.05), non-parametric or parametric tests were used. To investigate differences between the data collected during the first and second measurements of participants, the Student *t*-test was used. By use of the binary logistic regression model, it was examined which variables influenced the consumption of dietary supplements among adolescents in both genders and age groups. 

## 3. Results

**Figure 2:**
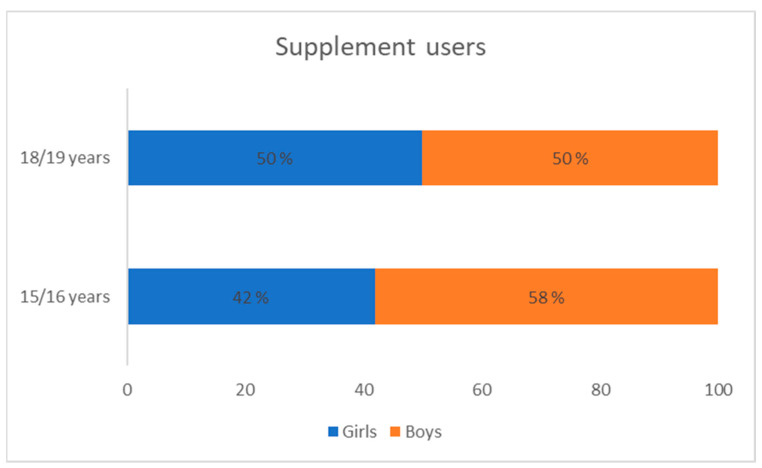
Share of dietary supplement users (girls and boys) during the first (15/16 years; *n* = 104 users) and second (18/19 years; *n* = 127 users) measurement.

A total of 607 respondents participated in the research—305 respondents were female (50.2%), while 302 respondents were male (49.8%). With the change in age, there was an increase in the number and share of dietary supplement users among the entire observed population. In addition, there was a change in the ratio of dietary supplement users by gender. At the age of 15/16, there was a higher percentage of boys (dietary supplement users) compared to girls (dietary supplement users). On the other hand, at the age of 18/19, the percentage between boys and girls who were dietary supplement users was equal, which is evident from Figure 2. Furthermore, by conducting a non-parametric test, a statistically significant difference in the number of girls who use dietary supplements was confirmed according to age.

**Figure 3:**
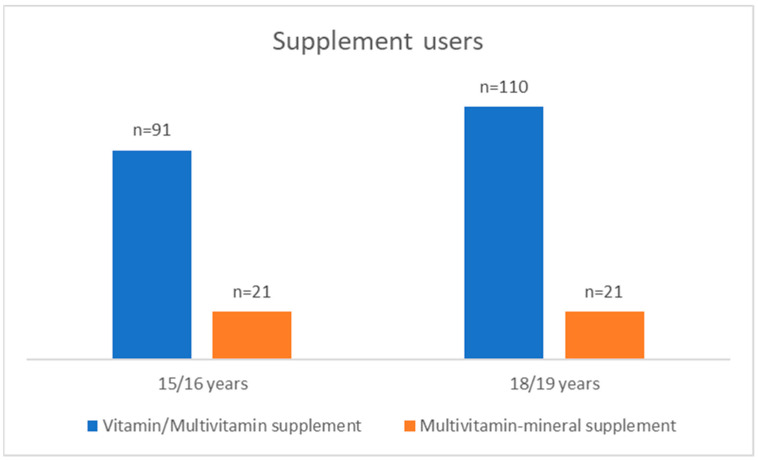
Distribution of dietary supplement users according to the type of preparations consumed during the first (15/16 years; *n* = 104 users) and second (18/19 years; *n* = 127 users) measurement.

Dietary supplement users were divided into two groups: (1) users who consumed one or more vitamins (including Cedevita) and (2) users who consumed multivitamin-mineral preparations (including sports and energy drinks). The users of the two groups, from both measurements, are shown in Figure 3. Changing age led to an increase in the intake of vitamin and multivitamin supplements, while the intake of multivitamin–mineral supplements remained the same. In addition, it should be pointed out that some supplement users consumed both supplements: vitamin/multivitamin and multivitamin–mineral (6.7% at age 15/16 year and 3.2% at the age 18/19 years). 

The most commonly used dietary supplement that contained only vitamins at the age of 15/16 was vitamin C, and it was consumed more by boys (58.3%). Cedevita as an enriched multivitamin preparation was consumed by 66.1% of users, and it was used more by boys (58.1%) than by girls (41.9%). When we look at the intake of vitamins and minerals through dietary supplements, the respondents took the most multivitamin–mineral preparations (without emphasizing which combinations were used). At the age of 18/19, there was a jump in the intake of vitamins of the B group, which were now consumed by 10.7% of users of dietary supplements. The beginning of vitamin D consumption was also observed (3.8% users). In the intake of both vitamins, we also observed a statistically significant difference—this was for the intake of B group vitamins (*p* = 0.001) and vitamin D (*p* = 0.03). The intake of Cedevita was decreasing, with age change, and it was consumed more by girls than by boys. The results are presented in Table 1.

The age of all subjects, along with anthropometric parameters and moderate-to-vigorous physical activity, are shown in Table 2. During the first and second measurement, the average age of girls, for dietary supplement users, was slightly lower than in non-users. The same case was with boys during the first measurement, while, in the second measurement, the age of users and non-users was the same. At the age of 15/16, moderate-to-vigorous physical activity, body mass, body mass index, and the sum of the thickness of four skin folds were higher in girls and boys for dietary supplement non-users (with the exception of moderate-to-vigorous physical activity in boys, for dietary supplement users, which had higher values than non-users). A statistically significant difference was observed for body mass index in girls of the same age between dietary supplement users and non-users (*p* = 0.004). At the age of 18/19, girls, for dietary supplement non-users, and boys, for dietary supplement users, had higher values of all the mentioned parameters (with the exception of the body mass index in boys, where the values were the same for users and non-users).

**Figure 4:**
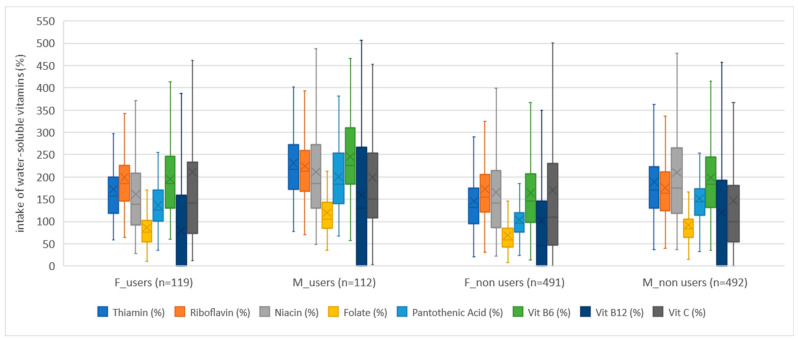
Percentage of water-soluble vitamin intake of dietary supplement users and non-users by gender, expressed as a percentage of the reference values of USDA recommendations. The number of girls and boys, dietary supplement users and non-users, is shown in brackets. Box plots are percentiles (Q1—lower quartile, Q3—upper quartile), median values, and mean values.

**Figure 5:**
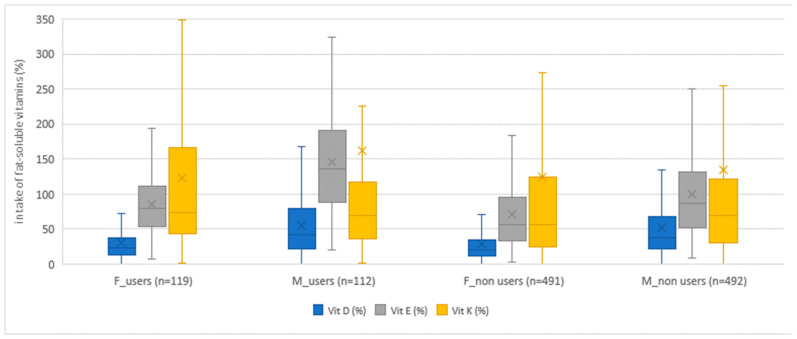
Percentage of fat-soluble vitamin intake of dietary supplement users and non-users by gender, expressed as a percentage of the reference values of USDA recommendations. The number of girls and boys, dietary supplement users and non-users, is shown in brackets. Box plots are percentiles (Q1—lower quartile, Q3—upper quartile), median values, and mean values.

**Figure 6:**
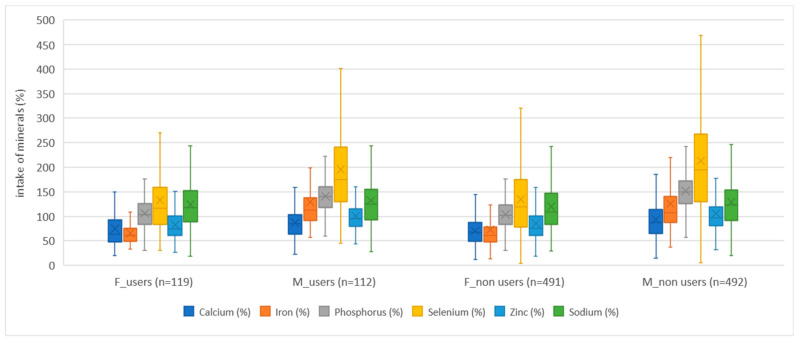
Percentage of mineral intake of dietary supplement users and non-users by gender, expressed as a percentage of the reference values of USDA recommendations. The number of girls and boys, dietary supplement users and non-users, is shown in brackets. Box plots are percentiles (Q1—lower quartile, Q3—upper quartile), median values, and mean values.

Dietary supplement users (both girls and boys) consumed more vitamins than non-users in both age groups when we monitored vitamin intake only from food. An exception in this study was the intake of niacin in girls in both age groups, where non-users had a higher average achieved intake of niacin, with statistically significant difference between users and non-users of dietary supplements. Apart from niacin, the exceptions were the achieved intakes for vitamin D at the age of 15/16 years and for vitamin K at the age of 18/19 years, where girls, non-users, had a higher average achieved intake, but without statistical significance. In boys, the average achieved intake was higher in dietary supplement users, with the exception of the average achieved intake of vitamin K and B_12_ at the age of 15/16 years, and the intake of niacin at the age of 18/19 years. In addition, boys at the age of 18/19 had the same average intake for vitamins B_12_ and D. As a rule, dietary supplement users had a smaller share of respondents who did not achieve the recommended intake of vitamins, which followed the USDA recommendations. The exceptions to the above were girls, dietary supplement non-users, whose share of respondents, who did not achieve the recommended intake, was lower for niacin and vitamin D, and boys, dietary supplement non-users, for vitamin K intake at the age of 15/16. Also, an exception was found in boys, dietary supplement non-users, aged 18/19, whose share of respondents, who did not achieve the recommended intake, was lower for niacin and vitamin K. The results are presented with the help of box–whisker diagrams (Figure 4, Figure 5 and Figure 6). The diagrams present compliance with the recommended daily intake of vitamins (Figure 4 and Figure 5) and minerals (Figure 6), thus indicating similarities/differences between dietary supplement users and non-users by gender for both age groups (together, not individually). The comparison between dietary supplement users and non-users according to gender, for water-soluble vitamins, can be seen in Figure 4, while, for fat-soluble vitamins, the comparison is shown in Figure 5. 

In the Supplemental Materials (Tables S1–S9), we additionally presented detailed results regarding comparisons between dietary supplement users and non-users according to gender and age (Tables S1 and S2). The tables also show statistically significant differences between dietary supplement users and non-users of the same gender and age. Statistically significant differences between girls who were users and who were not users of dietary supplements were visible for vitamins: thiamine (*p* = 0.02), riboflavin (*p* = 0.01), niacin (*p* = 0.02), pantothenic acid (*p* < 0.001), and folate (*p* = 0.03) at the age of 15/16 years; there were also differences for riboflavin (*p* = 0.04), pantothenic acid (*p* < 0.001), vitamin B_6_ (*p* = 0.005), vitamin E (*p* < 0.001), and folate (*p* = 0.01) at the age of 18/19 years. In boys, statistically significant differences were found between users and non-users of dietary supplements for the intake of thiamine (*p* < 0.001), riboflavin (*p* < 0.001), pantothenic acid (*p* < 0.001), vitamin B_6_ (*p* < 0.001), vitamin E (*p* < 0.001), folate (*p* < 0.001), and vitamin B_12_ (*p* = 0.02) at the age of 15/16 years; there were differences for thiamine (*p* = 0.01), riboflavin (*p* < 0.001), pantothenic acid (*p* = 0.004), vitamin C (*p* = 0.01), vitamin E (*p* < 0.001), and folate (*p* = 0.005) at the age of 18/19 years. The Supplementary Materials (Table S3) provide a detailed explanation of the vitamin intake of dietary supplement users and non-users following EFSA’s recommendations. The results follow the results of the USDA recommendations. Exceptions were found at the age of 15/16 in girls, dietary supplement non-users, for niacin intake, where non-users had a higher average achieved intake. For niacin, the share of respondents who consumed less than the recommended intake was higher in girls, for dietary supplement users. Additionally, exceptions were found in boys, dietary supplement non-users, for vitamin K intake, where non-users had a higher average achieved intake, and the users had a higher proportion of respondents who ingested less than the recommended intake. At the age of 18/19, for niacin intake, boys and girls, dietary supplement non-users, had a higher average intake. 

The intake of minerals in dietary supplements non-users (Tables S4–S6, including girls and boys) for both age groups was mostly higher than in dietary supplement users. In girls, for dietary supplement non-users in both age groups, we observed a higher average achieved intake of iron and zinc, while girls, dietary supplement users, in both age groups had a higher average achieved intake of calcium. The average achieved sodium intake was higher in girls, dietary supplement non-users, at the age of 15/16 and in dietary supplement users at the age of 18/19. On the other hand, in boys, dietary supplement non-users, in both age groups, we observed a higher average intake of calcium, phosphorus, and zinc. Boys, dietary supplement users, in both age groups, had a higher average iron intake. The average achieved sodium intake was higher in boys, dietary supplement non-users, at the age of 15/16 and in boys, dietary supplement users, at the age of 18/19. When we talk about the percentage of respondents who did not meet the recommended daily intake, we can say that result was almost equal. At the age of 15/16, girls, dietary supplement users achieved a higher percentage of the recommended daily intake for calcium, phosphorus, and zinc, while a higher percentage of male users achieved the recommended daily intake for iron and selenium. On the other hand, at the age of 18/19, girls, dietary supplement users achieved a higher percentage of the recommended daily intake for calcium, selenium, and sodium, while a higher percentage of male users, of the same age, achieved the recommended daily intake for iron, phosphorus, and sodium. The results are presented with the help of a box–whisker diagram. The diagrams (Figure 4, Figure 5 and Figure 6) show differences/comparison between dietary supplement users and non-users by gender for both age groups (together, not individually). The comparison between dietary supplement users and non-users according to gender, for minerals, can be seen in Figure 6. Additionally, in the Supplemental Materials, we presented detailed results and comparisons between dietary supplement users and non-users according to gender and age (Tables S4 and S5). The tables also show statistically significant differences between dietary supplement users and non-users of the same gender and age. A statistically significant difference was found in girls (between dietary supplement users and non-users) for iron (*p* = 0.005) at the age of 15/16 years. In boys, a statistically significant difference was found for phosphorus intake at the age of 18/19 years (*p* = 0.04). Table S6 shows the results obtained by comparing mineral intake with the EFSA’s recommendations. The results obtained following the EFSA recommendations show that the recommended achieved intake was higher in dietary supplement users in both genders and both age groups with two exceptions. For girls at the age of 15/16, dietary supplement non-users had a higher achieved average iron intake. For boys at the age of 18/19 dietary supplement non-users achieved a higher average intake for selenium. 

From the results shown in Table 3, it is visible that neither girls nor boys met the recommended intake of fruits and vegetables, in any age group, following the average achieved intake (g/day), regardless of whether they were dietary supplement users or non-users. In addition, fruit and vegetable intake decreased with age in the entire examined population. Girls, dietary supplement users, and boys, dietary supplement non-users, consumed more fruits and vegetables (following g/day) at the age of 15/16. At the age of 18/19, girls, dietary supplement non-users, and boys, dietary supplement users, had a higher average intake (g/day). On the other hand, following the relative-to-energy intake of fruits and vegetables, we observed that dietary supplement non-users in both gender and both age groups had higher values than users. The situation was similar with carbonated sweetened drinks, with the exception of girls, dietary supplement users, aged 18/19, who had higher values than non-users. The intake of non-carbonated sweetened drinks was higher among dietary supplement users in both genders and both age groups. The intake of fast food among girls, dietary supplement users, and boys, dietary supplement non-users, was higher in both age groups.

### Results of Logistic Regression

Logistic regression was used to compare the following quantitative variables: (1) body mass (kg); (2) body mass index (kg/m^2^); (3) sum of thickness of four skin folds (mm); (4) energy expenditure (kcal/kg/day); (5) intake of micronutrients (mg/1000 kcal or µg/1000 kcal); and (6) intake of fruits and vegetables (g/1000 kcal), fast food (g/1000 kcal), and sweetened beverages (g/1000 kcal); they also included the intake of vitamin, multivitamin, and multivitamin–mineral supplements among both genders for both age groups. Variables such as body mass, pantothenic acid, vitamin B_6_, folate, and non-carbonated sweetened beverages significantly contributed to the consumption of VMV dietary supplements for both genders at the age of 15/16 years. All variables had a 0.25% higher probability of contributing to the consumption of preparations of the same category and were also positively correlated. Pantothenic acid, vitamin B_6_, folate, and non-carbonated beverage sweeteners significantly contributed to the intake of preparations of the same category for both genders at the age of 18/19 years, had a 0.25% higher probability of contributing to their consumption, and were also positively correlated. Riboflavin significantly contributed to consumption and had a 0.25% higher probability of contributing to the consumption of MMV preparations at the age of 15/16 for both genders. Body mass index, although having a statistically significant contribution to the consumption of VMV dietary supplements, was not positively correlated with their intake for the age of 15/16 years for both genders. The same applied to the intake of niacin and the intake of fruits and vegetables. All results are presented in Supplemental Material Table S9.

## 4. Discussion

The aim of this work was to examine the change in the intake of vitamin and mineral-vitamin supplements, the change in the overall diet quality, and the changes in the intake of fruits, vegetables, fast food, and sweetened beverages of dietary supplement users and non-users regarding adolescents from 14 secondary schools in the City of Zagreb (Croatia) in a longitudinal study over 3 years. When monitoring the changes in diet quality that occurred with the age change, it should be considered that both measurements on the same subjects were carried out during adolescence. As part of the research, the age and anthropometry of dietary supplement users and non-users were presented in order to show the difference in the parameters of adolescents who used compared to those who did not use dietary supplements. The share of dietary supplement users and the most frequently used dietary supplements by adolescents are also shown. The main emphasis in this research was put on the assessment of the diet quality between users and non-users of dietary supplements. Therefore, the intake of micronutrients, fruits, vegetables, sweetened drinks, and fast food was determined in users and non-users of dietary supplements. A logistic regression was performed with the aim of predicting potential variables that could contribute to the intake of dietary supplements among adolescents.

Research shows that age change led to an increase in the number and share of dietary supplements users, especially vitamin and multivitamin preparations. Additionally, the change in age led to an increase in the number and share of girls for dietary supplement users. In this study, vitamin C was the most commonly used dietary supplement in both age groups, while, at the age of 15/16, it was consumed more by boys (if the consumption of Cedevita is not considered as a fortified multivitamin preparation). A similar result was observed in the EsKiMo II study. In this study, the largest share of dietary supplement users consumed vitamin C (43.9%) [8]. It is interesting to note that vitamin D (whose intake is least satisfied through diet) was not consumed by any user of dietary supplements at the age of 15/16, while, for the EsKiMo II study, vitamin D was consumed by 41.1% of respondents [8]. On the other hand, the change in age showed a decrease in the number of users of sports drinks, but it also showed an increase in the number of users of energy drinks, but only among boys. The intake of Cedevita, as a widely consumed vitamin fortified drink in Croatia, decreased with age, and consumption at the age of 18/19 was more common among girls than boys. Although Cedevita contains 50% of the recommended daily dose of several vitamins, the main problem with the consumption of this drink is the intake of added sugars (Tables S7 and S8). The original formulation of the drink contained 6–7% of added sugars in the prepared drink (mixed with water), but today’s formulation contains half of the original values. In the new formulation, the added sugars were replaced by *steviol glycoside.* In addition, the research shows that the number of Cedevita users decreased with age. However, what is more important is the contribution to the total intake of vitamins in dietary supplement users. Although the number of users decreased with age, we observed a greater contribution of vitamins from Cedevita to the total vitamin intake in dietary supplement users for thiamin, riboflavin, folic acid, pantothenic acid, vitamins B_6_, B_12_, C, and E for both genders and for niacin in boys. The results can be seen in the Supplemental Material Tables S7 and S8. Unfortunately, at the level of Croatia and Southeast European countries, we did not find similar research (previously conducted) on this topic due to their deficiency. However, a Canadian study was conducted on a sample of respondents aged 14 to 50 in 2021. It found that voluntarily fortified food in Canada significantly contributed to the total intake of micronutrients for five to seven tested nutrients (niacin, riboflavin, vitamin B_6_, vitamin B_12_, and zinc). It is important to note that the Canadian study included energy drinks, fortified drinks, cereals, and energy bars as voluntarily fortified foods [24], whereby we see that the Canadian study included more fortified foods in its research than the amount included in our study. In addition, a Dutch study states that voluntarily fortified food contributed from 9 to 78% to the total daily intake of vitamins and minerals, of course, in voluntarily fortified food consumers. Intakes of vitamins A, B_1_, B_2_, B_3_, B_6_, B_12_, C, D, E, calcium, iron, and folate equivalents were higher in users of voluntarily fortified food than in non-users. It should be noted that, in this study, the voluntary fortified foods consumed were mostly within the groups ‘Fats and oils’, ‘Non-alcoholic drinks,’ and ‘Dairy products and substitutes’ [25].

If we observe the nutrition quality between dietary supplement users and non-users, following the intake of vitamins and minerals only from food, we can conclude that dietary supplement users have a better diet quality due to the higher average achieved intake of most vitamins and minerals. This was also confirmed by the French study ‘NutriNet-Sante’, which states that dietary supplement users had a better diet (according to the intake of micronutrients) than non-users [26]. On the other hand, the NHANES study conducted from 2009–2012, on a sample of the population older than 2 years, showed that the intake of micronutrients through dietary supplements had a smaller impact on meeting the estimated average requirement (estimated average requirement, EAR) than food fortification [27]. Our results also follow the results of the Danish study, where it was observed that women who were also dietary supplement users, at the age of 18 and older, had a significantly higher intake of all micronutrients except niacin, which was also the case in our study [28]. The nutrient whose recommended intake was not met by both users and non-users of dietary supplements (in both genders and both age groups) was vitamin D. The unmet recommended intake for vitamin D in both genders and among all age groups was also visible in the Danish study [28]. Comparing the obtained data with other European countries, for the intake of vitamin D, we observe that, in Slovenia, the average intake of vitamin D among adolescents was 2.73 µg/day (including both genders), and it should be noted that the data for this research were collected through a Slovenian national survey on food consumption, which included a general questionnaire, two 24 h recalls, and a questionnaire on food preferences [29]. In Spain, a Mediterranean country, which is similar to Croatia, the average intake of vitamin D among girls aged 11 to 17 was 1.5 µg/day, and, in Poland, it was 3.2 µg/day. The average intake of vitamin D in boys of the same age ranged from 1.9 µg/day in France to 4.8 µg/day in Poland [30]. In addition, the HELENA study, which was conducted in 2012, stated that the intake of potassium, phosphorus, magnesium, zinc, and copper in adolescents of both genders was adequate, while the intake of sodium was even five times higher than recommended [12]. In our research, it is notable that girls (users and non-users) in both age groups, to the greatest extent, did not meet the recommended intake of iron and calcium (according to USDA recommendations), while boys (users and non-users), to the greatest extent, did not meet the recommended intake of calcium and zinc (according to USDA recommendations). The average sodium intake in both genders and both age groups was slightly higher than the recommended daily intake according to USDA recommendations.

The recommended intake of fruits and vegetables according to the WHO and the Food and Agriculture Organization of the United Nations (FAO) was 400 g or more per day [31]. It is evident from the research that our respondents, regardless of whether they were dietary supplement users or non-users, in both genders and both age groups, did not meet this intake. In fact, the intake was almost twice less than the recommended amount, which is in accordance with the HELENA study conducted on adolescents in Europe, where it was confirmed that adolescents ate only half of the recommended amount of fruits and vegetables [12]. Inadequate fruit consumption seems to be reported among adolescents on a global level [32]. Specifically, inadequate fruit consumption among Croatian adolescents could be partly explained by relatively high cost per 1000 kcal from fruit, in comparison with other food groups, as shown in a report for the Croatian market [33]. Fruit and vegetable intake decreased with age in both genders, following the average daily intake (g/day) and relative-to-energy intake (g/1000 kcal). The intake of non-carbonated sweetened beverages was higher in dietary supplement users in both genders and in both age groups, where it should be kept in mind that Cedevita was also included in this calculation. Although it is a drink that contributes to vitamin intake, we must not ignore it because of the sugar content. On the other hand, for example, in Switzerland, Van der Horst and Siergrist state that dietary supplement users consumed significantly less sweetened beverages than non-users [34]. The intake of fast food among girls, dietary supplement users, and boys, dietary supplement non-users, was higher in both age groups, while, for example, in the Canadian population older than 2 years, the intake of fast food was higher among non-users of dietary supplements [35]. 

The results of the logistic regression show that body mass, vitamins, pantothenic acid, vitamin B_6_, and folate, and non-carbonated sweetened drinks as quantitative variables significantly contributed to the consumption of VMV dietary supplements for both genders at the age of 15/16 years. As age changed, quantitative variables such as previously mentioned—vitamins and sweetened drinks—significantly contributed to the consumption of VMV preparations. On the other hand, riboflavin as a quantitative variable significantly contributed to the consumption of MMV preparations at the age of 15/16 years for both genders. The obtained results are partially correlated with the results of the EsKiMo II study, which showed that the variables body weight and physical activity were independent variables for the use of dietary supplements in adolescence [8]. Additionally, Van der Horst and Siegrist found positive correlation in the Swiss population older than 20 years between the consumption of ready-made food (fast food) and dietary supplements, while a negative correlation was found between the consumption of sweetened beverages and dietary supplements [34].

## 5. Limitation and Future Directions

When interpreting the results, it is necessary to additionally emphasize which food method was used to obtain the data (24 h recall) and that, for the purposes of the research, data on food and drink intake during one working day was collected. If a different dietary method (e.g., FFQ) had been used instead of a 24 h recall and we had been able to collect data for at least one or two more weekdays (or an additional weekend), the results might have been different and/or more complete. Additionally, we labeled adolescent as a supplement (non)user based on the use on a single day, although this limitation is shared with other studies having similar aims. In our study, we have tried to improve the recall of dietary supplement use by encouraging, when necessary, post interview contact with a subject by phone or e-mail with the aim to provide additional information on the reported supplement. In addition, when analyzing such data as were available in this study, it is possible to produce different conclusions depending on the definition of a supplement user, a definition of supplement itself, and the selection of dietary parameters, their definitions, and cut-off values discerning diets of a various quality. In addition, it should be emphasized that Cedevita, although its composition contributes to the daily intake of vitamins, is also rich in added sugars, and, as such, we included it in the category of non-carbonated sweetened drinks. However, today’s formulation of Cedevita has, when we follow the proportion of added sugars, a better composition (the proportion of sugar has been reduced by half compared to the original formulation). However, it must be kept in mind that the intake of non-carbonated sweetened drinks is higher among users of dietary supplements in both genders and both age groups. If we had not included Cedevita in this group, the question is whether the results would have been different. Also, at the time of conducting both measurements, our subjects were in adolescence. We are aware that today, children are entering puberty earlier more and more often. Therefore, our adolescents at the age of 18/19 may, for this very reason, have some characteristics of the adult group, but such a conclusion definitely needs to be further investigated in the future. 

Future studies on this topic should capture time trends in the prevalence of use and popularity of a specific supplement, and these results, in conjunction with national data for nutritional status, including intake, could serve as a basis for creating nutritional interventions in this pivotal age group.

## 6. Conclusions

The results of the CRO-PALS longitudinal study showed that, as the adolescents aged, an increase in the consumption of dietary supplements occurred in both genders, with an increase in the intake of VMV preparations. As a rule, girls who used dietary supplements had a lower body weight, body mass index, and the sum of the thickness of four skin folds in both age groups. The same applied to boys, dietary supplement users, but only at the age of 15/16. In general, dietary supplements users had a higher intake of most vitamins and minerals examined (here, the intake was from food only). The superior diet quality was achieved by girls, dietary supplement non-users, in both age groups, and by boys, dietary supplement non-users at the age of 18/19, with a higher intake of fruits and vegetables and a lower intake of fast food and sweetened drinks, in contrast to users of dietary supplements. 

## Figures and Tables

**Table 1:** Number and share of vitamin and multivitamin–mineral supplement users during the first (15/16 years) and second (18/19 years) measurement. Additionally, statistically significant differences (α = 0.05) are bolded.

	Vitamin and Multivitamin–Mineral Supplement Users				
Type of Dietary Supplement	1st Measurement (15/16 Years) Frequency		2nd Measurement (18/19 Years) Frequency		Statistically Significant
	N	%	N	%	*p*-Value
Vitamin C	12	10.7	17	13	0.3
Multivitamin	5	4.5	22	16.8	**0.001**
Beta carotene	1	0.9	1	0.8	1
B group vitamins	1	0.9	14	10.7	**0.001**
Vitamin D	0	0	5	3.8	**0.03**
Cedevita	74	66.1	61	46.6	0.2
Multivitamin–mineral	7	6.3	8	6.1	0.8
Iron with vitamins	3	2.7	5	3.8	0.5
Zinc with vitamins	2	1.8	0	0	0.2
Magnesium with vitamins	4	3.6	2	1.5	0.4
Calcium with vitamins	2	1.8	1	0.8	0.6
Sport drinks	5	4.5	3	2.3	0.5
Calcium and magnesium with vitamins	0	0	1	0.8	0.3
Energy drinks	0	0	2	1.5	0.2

**Table 2:** Average values with additional standard deviations and range indicated in the brackets for the following: age, anthropometric data, physical activity (moderate-to-vigorous physical activity) in girls and boys, dietary supplement users (n_girls 15/16 years_ = 44, n_boys 15/16 years_ = 60; n_girls 18/19 years_ = 75, n_boys 18/19 years_ = 52), and non-users (n_girls 15/16 years_ = 261, n_boys 15/16 years_ = 242; n_girls 18/19 years_ = 230, n_boys 18/19 years_ = 250). Results are presented as mean ± standard deviation (minimum; maximum). Additionally, a statistically significant difference between dietary supplement users and non-users was indicated (α = 0.05).

	Dietary Supplements Users (*n* = 104)		Dietary Supplement Non-Users (*n* = 503)	
1st measurement (15/16 years)				
	Girls	Boys	Girls	Boys
Age (years)	15.5 ± 0.3 (15.0;16.1)	15.6 ± 0.3 ^b^ (15.0;16.0)	15.6 ± 0.4 (14.4;16.9)	15.7 ± 0.4 ^b^ (15.0;16.9)
Body mass (kg)	57.2 ± 9.3 (41;84.7)	68.1 ± 13.7 (39;110.3)	59.7 ± 9.3 (39.6;100.3)	68.5 ± 12.3 (43.3;121.1)
Body mass index (kg/m^2^)	20.5 ± 2.5 ^a^ (16.8;27.6)	21.3 ± 3.9 (15.6;33.5)	21.7 ± 3.2 ^a^ (15.8;38.8)	21.7 ± 3.4 (15.8;34.8)
The sum of the skin fold thickness (mm)	46.6 ± 15.5 (24.6;93.6)	35.3 ± 19.1 (18;102.6)	50.7 ± 15.8 (24.2;145)	37.1 ± 17.6 (16.2;104.6)
Moderate-to-vigorous physical activity (kcal/kg/day)	8.2 ± 5.4 (0.4;22.2)	11.6 ± 7.4 (0.0;32.9)	9.1 ± 6.2 (0.0;40)	10.7 ± 6.5 (1.2;33.9)
	**Dietary Supplements Users** **(*n* = 127)**		**Dietary Supplement Non-Users** **(*n* = 480)**	
2nd Measurement (18/19 Years)				
	Girls	Boys	Girls	Boys
Age (years)	18.5 ± 0.3 (17.8;19.7)	18.6 ± 0.4 (18.0;19.9)	18.6 ± 0.4 (17.2;19.9)	18.6 ± 0.4 (17.2;19.8)
Body mass (kg)	60.3 ± 8.5 (41;80.8)	74.5 ± 13.8 (49.4;122.9)	61.1 ± 9.7 (40.6;97.9)	74 ± 11.4 (52.4;116.6)
Body mass index (kg/m^2^)	21.6 ± 2.9 (17;32)	22.7 ± 3.6 (16.3;36.9)	22 ± 3.3 (16.4;39.8)	22.7 ± 3.1 (16.7;35.2)
The sum of the skin fold thickness (mm)	48.1 ± 15 (22.3;115.5)	37.1 ± 16 (22.3;97.6)	50.8 ± 17.1 (25.1;147.1)	36.6 ± 15.9 (15.9;122.3)
Moderate-to-vigorous physical activity (kcal/kg/day)	6.7 ± 4.3 (0.0;24.9)	9.8 ± 6.3 (1.0;32)	7.8 ± 5.8 (0.0;31.6)	9.0 ± 5.3 (0.0;28.5)

^a^—statistically significant difference for the same observed category, between girls, dietary supplement users and non-users, of the same age; ^b^—statistically significant difference for the same observed category, between boys, dietary supplement users and non-users, of the same age.

**Table 3:** Average values with additional standard deviations and range indicated in the brackets for intake of fruits and vegetables, fast food, sweetened beverages in girls and boys, users (n_girls 15/16 Years_ = 44, n_boys 15/16 Years_ = 60; n_girls 18/19 Years_ = 75, n_boys 18/19 Years_ = 52), and non-users (n_girls 15/16 Years_ = 261, n_boys 15/16 Years_ = 242; n_girls 18/19 Years_ = 230, n_boys 18/19 Years_ = 250) of dietary supplements in both age groups. Results are presented as mean ± standard deviation (minimum; maximum). Additionally, statistically significant differences (α = 0.05) are indicated.

Intakes for Dietary Supplement Users and Non-Users in Both Age Groups				
1st measurement (15/16 Years)				
	Dietary supplement users (*n* = 104)		Dietary supplement non-users (*n* = 503)	
	Girls	Boys	Girls	Boys
Fruit and vegetables (g/day)	268.9 ± 414 (0;1700)	292.1 ± 362.2 (0;1540)	241.3 ± 271.1 (0;1400)	312.2 ± 398.6 (0;3510)
Fruit and vegetables (g/1000 kcal)	139.9 ± 201.2 (0;779.7)	98.4 ± 104.2 (0;370.9)	153.7 ± 166.6 (0;1005.4)	125.5 ± 134.1 (0;754.8)
Fast food (g/1000 kcal)	90.4 ± 104.6 (0;400.3)	91.7 ± 69.7 (0;292.8)	87.6 ± 111.6 (0;914.4)	93.7 ± 86.6 (0;493.7)
Non-carbonated sweetened drinks (g/1000 kcal)	134.1 ± 244.4 (0;1491.4)	146.3 ± 177.5 ^a^ (0;1001.5)	86.4 ± 177.7 (0;1344.0)	80.2 ± 138.2 ^a^ (0;810.2)
Carbonated sweetened drinks (g/1000 kcal)	32.6 ± 86.7 (0;385.0)	43.0 ± 78.2 (0;333.8)	43.1 ± 165.4 (0;2135.1)	56.0 ± 120.0 (0;935.3)
2nd measurement (18/19 Years)				
	Dietary supplement users (*n* = 127)		Dietary supplement non-users (*n* = 480)	
	Girls	Boys	Girls	Boys
Fruit and vegetables (g/day)	202.6 ± 238.7 (0;1220)	234.5 ± 321.1 (0;1710)	216.8 ± 247.4 (0;1215)	229.4 ± 273.5 (0;1270)
Fruit and vegetables (g/1000 kcal)	134.0 ± 160.8 (0;645.2)	83.7 ± 107.1 (0;562.2)	141.8 ± 157.3 (0;762.4)	94.3 ± 116.3 (0;853.9)
Fast food (g/1000 kcal)	92.8 ± 103.1 (0;429.5)	109.1 ± 82.4 (0;319.2)	76.8 ± 86.3 (0;432.1)	114.5 ± 108.2 (0;1000.0)
Non-carbonated sweetened drinks (g/1000 kcal)	94.8 ± 153.0 ^b^ (0;849.5)	71.8 ± 89.7 (0;308.0)	50.4 ± 114.2 ^b^ (0;791.9)	44.9 ± 105.2 (0;850.3)
Carbonated sweetened drinks (g/1000 kcal)	32.2 ± 81.6 (0;498.1)	41.8 ± 83.1 (0;394.1)	29.6 ± 94.6 (0;791.9)	46.3 ± 110.6 (0;850.3)

^a^—identification of statistically significant difference, for the same observed category, between boys’ dietary supplement users and non-users, of the same age; ^b^—identification of statistically significant difference for the same observed category, between girls’ dietary supplement users and non-users, of the same age.

## Data Availability

The data presented in this study are available upon request from the corresponding author.

## Supplementary Materials

The following supporting information can be downloaded at: https://www.mdpi.com/article/10.3390/nu15122783/s1: Table S1: The average achieved intake of vitamins in girls and boys (dietary supplement users and non-users) at the age of 15/16 years; Table S2: The average achieved intake of vitamins in girls and boys (dietary supplement users and non-users) at the age of 18/19 years; Table S3: The average achieved vitamin intake according to EFSA recommendations for girls and boys, dietary supplement users and non-users, in both age groups; Table S4: The average achieved intake of minerals in girls and boys (dietary supplement users and non-users) at the age of 15/16 years; Table S5: The average achieved intake of minerals in girls and boys (dietary supplement users and non-users) at the age of 18/19 years; Table S6: The average achieved mineral intake according to EFSA recommendations for girls and boys, dietary supplement users and non-users, in both age groups; Table S7: Energy and nutritional value of Cedevita instant drink per 100 g and 19 g (recommended daily dose) of powder; Table S8: Contribution of vitamins from Cedevita to the total intake of vitamins in dietary supplement users (girls and boys) in both age groups; Table S9: Results obtained by logistic regression taking into account quantitative variables on the binary response of the consumption of vitamin, multivitamin, and multivitamin–mineral preparations for both genders and in both age groups.

## References

[1] EFSA—European Food Safety Authority Food Supplement. https://www.efsa.europa.eu/en/topics/topic/food-supplements.

[2] Dickinson A., MacKay D. (2014). Health habits and other characteristics of dietary supplement users: A review. Nutr. J..

[3] Dickinson A., MacKay D., Wong A. (2015). Consumer attitudes about the role of multivitamins and other dietary supplements: Report of a survey. Nutr. J..

[4] Mazure M., Mól N., Zasada M., Zasada W., Pyznar O., Kwinta P. (2022). Dietary supplements use among children from south-eastern Poland. Pediatr. Pol.-Pol. J. Paediatr..

[5] Pavičić Ž.S., Tomljanović A., Kenđel J.G., Krešić G., Cvijanović P.O., Dragaš-Zubalj N., Prokurica I.P. (2018). Prevalence, Knowledge and Attitudes Concerning Dietary Supplements among a Student Population in Croatia. Int. J. Environ. Res. Pub Health.

[6] Jun S., Cowan A.E., Tooze J.A., Gahche J.J., Dwyer J.T., Eicher-Miller H.E., Bhadra A., Guenther P.M., Potischman N., Dodd K.W. (2018). Dietary Supplement Use among U.S. Children by Family Income, Food Security Level, and Nutrition Assistance Program Participation Status in 2011–2014. Nutrients.

[7] Panjwani A.A., Cowan A.E., Jun S., Bailey R.L. (2021). Trends in Nutrient and non-Nutrient containing Dietary Supplement Use amond U.S. Children from 1999–2016. J. Pediatr..

[8] Perlitz H., Mensink G.B.M., Lage B.C., Richter A., Brettschneider A.K., Lehmann F., Patelakis E., Frank M., Heide K., Haftenberger M. (2019). Use of vitamin and mineral supplements among adolescents living in Germany-Results from EsKiMo II. Nutrients.

[9] Frey A., Hoffmann I., Heuer T. (2017). Characterisation of vitamin and mineral supplement users differentiated according to their motives for using supplements:results of the German National Nutrition Monitroing (NEMONIT). Pub Health Nutr..

[10] Ross A.C., Caballero B.H., Cousins R.J., Tucker K.L., Ziegler T.R., St-Onge M.P., Keller L.K. (2013). Nutrition in Adolescence. Modern Nutrition in Health and Diesease.

[11] Al Ani M.F., Al Subhi L.K., Bose S. (2016). Consumption of fruits and vegetables among adolescents: A multinational comparison of eleven conutries in the Eastern Mediterranean Region. Br. J. Nutr..

[12] Diethelm K., Jankovic N., Moreno L.A., Huybrechts I., De Henauw S., De Vriendt T., Gonzalez-Gross M., Leclercq C., Gottrand F., Gilbert C.C. (2011). Food intake of European adolescents in the light of different food-based dietary guidelines: Results of the HELENA (Healthy Lifestyle in Europe by Nutrition in Adolescence) Study. Public Health Nutr..

[13] Rešetar J., Pfeifer D., Mišigoj-Duraković M., Sorić M., Gajdoš J.K., Šatalić Z. (2020). Eveningness in Energy Intake among Adolescents with Implication on Anthropometric Indicators of Nutritional Status: The CRO-PALS Longitudinal Study. Nutrients.

[14] Wong S.L., Leatherdale S.T., Manske S.R. (2006). Reliability and Validity of a School-Based Physical Activity Questionnaire. Med. Sci. Sports Exerc..

[15] Moshfegh A.J., Rhodes D.G., Baer D.J., Murayi T., Clemens J.C., Rumpler W.V., Paul D.R., Sebastian R.S., Kuczynski K.J., Ingwersen L.A. (2008). The US Department of Agriculture Automated Multiple-Pass Method reduces bias in the collection of energy intakes. Am. J. Clin. Nutr..

[16] Senta A., Pucarin-Cvetković J., Doko J.J. (1990). Quantitative Models of Food and Meals. Postgraduate Student Manual.

[17] Kaić-Rak A., Antonić K. (1990). Tablice O Sastavu Namirnica I Pića.

[18] Danish Food Composition Databank. http://www.foodcomp.dk/.

[19] USDA—U.S. Department of Agriculture, Agricultural Research Service. FoodData Central. https://fdc.nal.usda.gov/.

[20] Vučetić M. (2013). Velika Knjiga Kuharstva.

[21] Sichert-Hellert W., Wenz G., Kersting M. (2006). Vitamin Intakes from Supplements and Fortified Food in German Children and Adolescents: Results from the DONALD Study. J. Nutr..

[22] Prynne C.J., Mishra G.D., O’Connell M.A., Muniz G., Laskey M.A., Yan L., Prentice A., Ginty F. (2006). Fruit and vegetable intakes and bone mineral status: A cross-sectional study in 5 age and sex cohorts. Am. J. Clin. Nutr..

[23] Lioret S., Volatier J.L., Lafax L., Touvier M., Maire B. (2009). Is food portion size a risk factor of childhood overweight?. Eur. J. Clin. Nutr..

[24] Tarasuk V., Brassard D. (2021). The effect of consuming voluntarily fortified food and beverages on usual nutrient intakes in the Canadian population. Food Nutr. Res..

[25] Jong M.H., Nawijn E.L., Verkaik-Kloosterman J. (2022). Contribution of voluntary fortified foods to micronutrient intake in The Netherlands. Eur. J. Nutr..

[26] Pouchieu C., Andreeva V.A., Peneau S., Kesse-Guyot E., Lassale C., Hercberg S., Touvier M. (2013). Sociodemographic, lifestyle and dietary correlates of dietary supplement use in a large sample of French adults: Results from the NutriNet-Sante’ cohort study. Br. J. Nutr..

[27] Newman J.C., Malek A.M., Hunt K.J., Marriott B.P. (2019). Nutrients in the US Diet: Naturally Occurring or Enriched/Fortified Food and Beverage Sources, Plus Dietary Supplements: NHANES 2009–2012. J. Nutr..

[28] Tetens I., Biltoft-Jensen A., Spagner C., Christensen T., Gille M.B., Bugel S., Banke Rasmussen L. (2011). Intake of micronutrients among Danish adult users and non-users of dietary supplements. Food Nutr. Res..

[29] Fidler Mis N., Kobe H., Stimec M. (2012). Dietary intake of macro- and micronutrients in Slovenian adolescents: Comparison with reference values. Ann. Nutr. Metab..

[30] Mensink G.B.M., Fletcher R., Gurinovic M., Huybrechts I., Lafay L., Serra-Majem L., Szponar L., Tetens I., Verkaik-Kloosterman J., Baka A. (2013). Mapping low intake of micronutrients across Europe. Br. J. Nutr..

[31] WHO—World Health Organization Diet, Nutrition and the Prevention of Chronic Diseases. https://apps.who.int/iris/handle/10665/42665..

[32] Heslin A.M., McNulty B. (2023). Adolescent nutrition and health: Characteristics, risk factors and opportunities of an overlooked life stage. Proc. Nutr. Soc..

[33] Bolarić M., Šatalić Z. (2013). The relation between food price, energy density and diet quality. Croat. J. Food Sci. Technol..

[34] Van der Horst K., Siegrist M. (2011). Vitamin and mineral supplement users. Do they have healthy or unhealthy dietary behaviours?. Appetite.

[35] Black J.L., Billette J.M. (2015). Fast food intake in Canada: Differences among Canadians with diverse demographic, socio-economic and lifestyle characteristics. Can. J. Public Health.

