# Supplementary material for: Assessment of Nutrient Intake and Diet Quality in Adolescent Dietary Supplement Users vs. Non-Users: The CRO-PALS Longitudinal Study
Source: Nutrients. 2023 Jun 17;15(12):2783. doi: 10.3390/nu15122783 (PMC10303724; doi:10.3390/nu15122783)
Supplement: Supplementary file 1 [file nutrients-15-02783-s001.zip › nutrients-2439830-supplementary.pdf]

nutrients MDPI

Table S1. The average achieved intake of vitamins in girls and boys who are dietary supplement users ($n_{girls\ 15/16\ Years}$ = 44, $n_{boys\ 15/16\ Years}$ = 60) and non-users ($n_{girls\ 15/16\ Years}$ = 261, $n_{boys\ 15/16\ Years}$ = 242) at the age of 15/16 years old. Results are presented as mean ± standard deviation (minimum; maximum). Additionally, statistically significant difference (α = 0.05) is indicated. The percentage of respondents (users and non-users of dietary supplements) who did not achieve the recommended daily intake according to USDA recommendations (with a tolerance of 10%) is also shown.

The average achieved intake of vitamins in dietary supplement users and non-users at the age of 15/16 years old					
Girls		Dietary supplement users (n = 44)		Dietary supplement non-users (n = 261)	
Vitamins	Recommended intake	Achieved intake	% below recommendations	Achieved intake	% below recommendations
Thiamine (mg/1000 kcal)	0.5	0.8±0.3[a] (0.3;1.7)	6.8	0.7±0.4[b] (0.2;3.3)	22.6
Riboflavin (mg/1000 kcal)	0.5	1.1±0.4[a] (0.5;2.8)	0	0.9±0.5[b] (0.2;3.3)	13.4
Niacin (mg/1000 kcal)	7.0	8.8±5.3[a] (2.1;24.7)	43.2	11.0±7.1[b] (1.5;40.8)	31.8
Pantothenic acid (mg/1000 kcal)	2.5	3.4±1.4[a] (0.9;8.4)	11.4	2.5±1.8[b] (0.6;20.4)	47.9
Vitamin B_6 (mg/1000 kcal)	0.6	1.0±0.5 (0.4;3.1)	2.3	0.9±0.6 (0.1;4.4)	24.9
Vitamin C (mg/1000 kcal)	33	85.5±101.6 (9.7;486.8)	31.7	70.3±65.7 (0.0;414.5)	41.9
Vitamin E (mg/1000 kcal)	8	6.2±2.8 (0.6;12.1)	65.9	5.8±4.9 (0.2;48.3)	71.6
Vitamin K (µg/1000 kcal)	28	37.9±44.7 (1.5;211.9)	56.8	29.4±40.2 (0.0;314.6)	63.1
Folate (µg/1000 kcal)	200	184.1±134.8[a] (30.7;781.1)	61.4	137.8±87.2[b] (15.9;646.2)	79.7
Vitamin B_{12} (µg/1000 kcal)	1.2	2.6±1.5 (0.7;7.5)	9.1	2.3±2.1 (0.1;14.4)	25.1
Vitamin D (µg/1000 kcal)	3	0.8±0.7 (0.0;4.2)	97.7	0.9±1.0 (0.0;8.6)	96.2
Boys		Dietary supplement users (n = 60)		Dietary supplement non-users (n = 242)	
Vitamins	Recommended intake	Achieved intake	% below recommendations	Achieved intake	% below recommendations
Thiamine (mg/1000 kcal)	0.4	0.9±0.4[c] (0.3;2.5)	1.7	0.7±0.3[d] (0.3;2.3)	3.7
Riboflavin (mg/1000 kcal)	0.5	1.1±0.4[c] (0.3;3.2)	1.7	0.9±0.4[d] (0.2;2.6)	7.0
Niacin (mg/1000 kcal)	5.7	11.6±5.9 (2.8;27.9)	13.3	10.7±6.2 (2.1;30.5)	15.7
Pantothenic acid (mg/1000 kcal)	1.8	3.8±1.7[c] (1.2;12.3)	3.3	2.6±1.0[d] (0.7;12.4)	8.3
Vitamin B_6 (mg/1000 kcal)	0.5	1.3±0.5[c] (0.5;3.1)	0	0.9±0.4[d] (0.2;3.2)	5.8
Vitamin C (mg/1000 kcal)	27	57.1±45.7 (4.2;252.0)	16.7	45.6±45.4 (0.9;269.7)	42.1
Vitamin E	5	7.8±4.5[c]	25	5.2±3.3[d]	50

(mg/1000 kcal)		(1.0;26.6)		(0.4;21.0)	
Vitamin K	23	23.0±28.8	65	30.6±68.3	58.4
(μg/1000 kcal)		(0.3;201.6)		(0.0;790.9)	
Folate	140	171.9±83.2^{c}	30	132.0±65.0^{d}	55.8
(μg/1000 kcal)		(50.0;1184.2)		(2.1;451.8)	
Vitamin B_{12}	0.9	2.7±1.4^{c}	0	3.6±1.4^{d}	6.6
(μg/1000 kcal)		(1.0;7.3)		(0.3;8.2)	
Vitamin D	2	1.1±1.0	83.3	0.9±0.8	83.4
(μg/1000 kcal)		(0.0;4.6)		(0.0;6.6)	

$^{ab-}$different letters for statistically significant difference between girls. dietary supplement users and non-users. of the same age. for the same observed category; ^{cd}-different letters for statistically significant difference between boys. dietary supplement users and non-users. of the same age. for the same observed category

Table S2. The average achieved intake of vitamins in girls and boys who are dietary supplement users ($n_{girls\ 18/19\ Years}$ = 75. $n_{boys\ 18/19\ Years}$ = 52) and non-users ($n_{girls\ 18/19\ Years}$ = 230. $n_{boys\ 18/19\ Years}$ = 250) at the age of 18/19 years old. Results are presented as mean ± standard deviation (minimum; maximum). Additionally. statistically significant difference (α = 0.05) is indicated. The percentage of respondents (users and non-users of food supplements) who did not achieve the recommended daily intake according to USDA recommendations (with a tolerance of 10%) is also shown.

The average achieved intake of vitamins in dietary supplement users and non-users at the age of 18/19 years old					
Girls		Dietary supplement users (n = 75)		Dietary supplement non-users (n = 230)	
Vitamins	Recommended intake	Achieved intake	% below recommendations	Achieved intake	% below recommendations
Thiamine (mg/1000 kcal)	0.5	0.9±0.5 (0.3;4.2)	9.3	0.8±0.3 (0.1;0.1)	20
Riboflavin (mg/1000 kcal)	0.5	1.0±0.4[a] (0.3;2.4)	1.3	0.8±0.4[b] (0.3;2.2)	7.8
Niacin (mg/1000 kcal)	7	12.7±8.2 (1.9;49.4)	13.3	19.0±13.6[b] (2.6;93.3)	20.9
Pantothenic acid (mg/1000 kcal)	2.5	3.4±1.4[a] (0.9;8.0)	21.3	2.6±1.1[b] (0.7;10.1)	35.6
Vitamin B_6 (mg/1000 kcal)	0.6	1.2±0.6[a] (0.4;4.3)	5.3	1.0±0.5[b] (0.1;3.3)	14.8
Vitamin C (mg/1000 kcal)	33	60.1±59.7 (3.8;324.5)	34.7	52.8±58.4 (0.0;363.1)	45.8
Vitamin E (mg/1000 kcal)	8	7.1±3.6[a] (1.0;17.8)	57.3	5.4±3.5[b] (0.6;15.4)	72.2
Vitamin K (µg/1000 kcal)	28	32.3±32.0 (0.6;165.6)	58.7	41.0±129.7 (0.0;1232.5)	66.4
Folate (µg/1000 kcal)	200	165.0±85.3[a] (20.6;487.6)	64	136.1±85.4[b] (18.3;609.1)	79.6
Vitamin B_{12} (µg/1000 kcal)	1.2	0.0±0.0 (0.0;0.0)	100	0.0±0.0 (0.0;0.0)	100
Vitamin D (µg/1000 kcal)	3	1.0±0.9 (0.0;4.4)	93.3	0.9±0.9 (0.0;6.6)	95.2
Boys		Dietary supplement users (n = 52)		Dietary supplement non-users (n = 250)	
Vitamins	Recommended intake	Achieved intake	% below recommendations	Achieved intake	% below recommendations
Thiamine (mg/1000 kcal)	0.4	0.9±0.3[c] (0.4;8727.3)	0	0.8±0.4[d] (0.1;2.6)	3.6
Riboflavin (mg/1000 kcal)	0.5	1.1±0.4[c] (0.5;2.2)	0	0.9±0.4[d] (0.2;3.8)	7.6
Niacin (mg/1000 kcal)	5.7	12.5±7.3 (3.1;36.0)	11.5	33.6±22.8 (1.6;135.6)	7.2
Pantothenic acid (mg/1000 kcal)	1.8	3.4±1.2[c] (1.6;6.9)	1.9	2.9±1.3[d] (0.6;13.7)	8
Vitamin B_6 (mg/1000 kcal)	0.5	1.2±0.6 (0.3;4.1)	1.9	1.0±0.5 (0.2;2.6)	8
Vitamin C (mg/1000 kcal)	27	50.0±43.2[c] (1.0;248.1)	23.1	33.9±33.9[d] (0.0;195.8)	49.2
Vitamin E (mg/1000 kcal)	5	6.7±3.0[c] (1.1;16.0)	25	4.8±3.2[d] (0.4;15.8)	56
Vitamin K	23	54.0±167.5	68	31.1±74.5	65.6

(µg/1000 kcal)		(1.0;880.0)		(0.0;821.5)	
Folate	140	161.9±84.9[c]	40.4	124.9±84.8[d]	61.6
(µg/1000 kcal)		(58.8;487.4)		(27.0;1102.1)	
Vitamin B_{12}	0.9	0.0±0.0	100	0.0±0.0	100
(µg/1000 kcal)		(0.0;0.0)		(0.0;0.0)	
Vitamin D	2	1.1±0.8	80.8	1.1±0.9	81.6
(µg/1000 kcal)		(0.3;3.1)		(0.0;5.2)	

[ab]-different letters for statistically significant difference between girls. dietary supplement users and non-users. of the same age. for the same observed category; [cd]-different letters for statistically significant difference between boys. dietary supplement users and non-users. of the same age. for the same observed category

Table S3. The average achieved intake of vitamins in girls and boys. dietary supplement users ($n_{girls\ 15/16\ Years}$ = 44. $n_{boys\ 15/16\ Years}$ = 60; $n_{girls\ 18/19\ Years}$ = 75. $n_{boys\ 18/19\ Years}$ = 52) and non-users ($n_{girls\ 15/16\ Years}$ = 261. $n_{boys\ 15/16\ Years}$ = 242; $n_{girls\ 18/19\ Years}$ = 230. $n_{boys\ 18/19\ Years}$ = 250) at both ages. Results are presented as mean±standard deviation (minimum; maximum). Additionally. statistically significant similarity or difference (α = 0.05) is indicated. The percentage of respondents (users and non-users of dietary supplements) who did not achieve the recommended daily intake according to EFSA recommendations (with a tolerance of 10%) is also shown.

The average achieved intake of vitamins in dietary supplement users and non-users at both ages					
1st measurement (15/16 Years)					
Girls		Dietary supplement users		Dietary supplement non-users	
	Recommended intake	Achieved intake	% under recommendations	Achieved intake	% under recommendations
Thiamine (mg)	0.76	1.6±0.9[a] (0.3;4.8)	9.1	1.1±0.8[a] (0.1;7.1)	26.8
Riboflavin (mg)	1.4	2.0±1.0[a] (0.3;5.1)	13.6	1.4±1.1[a] (0.1;9.1)	54.4
Niacin (mg)	13.7	17.0±12.9 (2.8;59.2)	47.7	18.0±14.1 (0.9;99.7)	41
Pantothenic acid (mg)	5	6.5±3.2[a] (0.9;15.5)	25	4.0±2.7[a] (0.5;26.6)	67.4
Vitamin B_6 (mg)	1.3	2.0±1.6[a] (0.6;7.6)	22.7	1.6±1.2[a] (0.1;9.3)	46
Vitamin C (mg)	75	151.3±172.9[a] (15.7;938.8)	34.9	93.2±116.3[a] (0.0;876.4)	56.8
Vitamin E (mg)	11	12.4±8.7[a] (0.6;46.1)	45.4	9.4±8.0[a] (0.1;43.0)	67
Vitamin K (μg)	65	70.7±100.8 (1.5;580.8)	59.1	47.6±72.6 (0.0;557.8)	77.4
Folate (μg)	250	338.7±246.1[a] (30.9;1493.8)	29.5	220.2±167.7[a] (7.1;1247.4)	65.1
Vitamin B_{12} (μg)	4	5.0±3.1[a] (0.3;14.2)	38.6	3.9±4.3[a] (0.1;30.2)	65.5
Vitamin D (μg)	15	1.7±1.9 (0.0;8.3)	100	1.4±1.7 (0.0;11.5)	100
Boys		Dietary supplement users		Dietary supplement non-users	
	Recommended intake	Achieved intake	% under recommendations	Achieved intake	% under recommendations
Thiamine (mg)	0.86	2.5±1.2[b] (0.4;6.8)	5	1.8±1.1[b] (0.4;10.1)	12
Riboflavin (mg)	1.4	3.1±1.6[b] (0.5;8.1)	6.7	2.2±1.3[b] (0.3;7.0)	21.5
Niacin (mg)	16.5	32.5±20.4 (3.9;95.8)	20	27.3±19.2 (2.5;98.1)	31.4
Pantothenic acid (mg)	5	9.9±4.4[b] (1.5;19.5)	8.3	6.5±3.6[b] (0.7;33.4)	28.9
Vitamin B_6 (mg)	1.5	3.4±1.6[b] (0.7;7.6)	5	2.4±1.4[b] (0.3;7.7)	26.9
Vitamin C (mg)	85	149.0±108.6[b] (5.6±479.4)	20	112.6±124.1[b] (1.2;978.3)	53.1
Vitamin E	13	21.2±12.7[b]	28.3	12.9±9.4[b]	56.2

(mg)		(1.8;48.9)		(0.5;51.1)	
Vitamin K (μg)	65	64.7±98.1 (0.8;732.0)	66.1	74.9±157.0 (0.0;1602.9)	65.5
Folate (μg)	250	453.1±217.4[b] (127.6;1184.2)	15	329.3±222.2[b] (2.6±1638.8)	34.7
Vitamin B_{12} (μg)	4	7.5±5.0[b] (1.3;26.0)	21.7	5.4±4.4[b] (0.3;34.7)	42.6
Vitamin D (μg)	15	3.3±3.8 (0.0;19.6)	96.7	2.3±2.1 (0.0;11.6)	100
2nd measurement (18/19 Years)					
Girls		Dietary supplement users		Dietary supplement non-users	
	Recommended intake	Achieved intake	% under recommendations	Achieved intake	% under recommendations
Thiamine (mg)	0.69	1.5±1.0[a] (0.3;6.4)	10.7	1.2±0.7[a] (0.1;3.7)	21.3
Riboflavin (mg)	1.3	1.6±0.8[a] (0.3;4.0)	33.3	1.3±0.7[a] (0.2;4.5)	53.5
Niacin (mg)	12.4	21.6±14.3 (2.9;77.5)	28	19.0±13.6 (2.6;93.3)	30.4
Pantothenic acid (mg)	5	5.7±3.0[a] (0.8;17.6)	41.3	4.1±2.3[a] (0.8;22.3)	65.2
Vitamin B_6 (mg)	1.3	2.1±1.0[a] (0.3;5.0)	17.3	1.6±1.0[a] (0.1;5.3)	39.1
Vitamin C (mg)	80	93.6±82.1 (3.8;434.4)	49.3	82.5±101.3 (0.0;739.9)	63.9
Vitamin E (mg)	11	11.8±6.8[a] (0.9;35.1)	46.7	8.5±6.7[a] (0.7;58.4)	64.3
Vitamin K (μg)	70	53.5±52.7 (0.6;262)	70.7	56.6±166.3 (0.0;1952.6)	79.7
Folate (μg)	250	266.7±134.4[a] (30.2;736.0)	44	206.1±129.6[a] (11.5;837.2)	70
Vitamin B_{12} (μg)	4	4.1±2.4 (0.3;10.9)	46.7	3.5±3.0 (0.0;17.9)	67.4
Vitamin D (μg)	15	1.6±1.6 (0.0;8.0)	100	1.4±1.4 (0.0;9.6)	100
Boys		Dietary supplement users		Dietary supplement non-users	
	Recommended intake	Achieved intake	% under recommendations	Achieved intake	% under recommendations
Thiamine (mg)	0.86	2.5±1.1[b] (0.7;6.1)	3.8	2.0±1.3[b] (0.3;7.7)	9.2
Riboflavin (mg)	1.3	2.9±1.2[b] (1.0;5.8)	1.9	2.3±1.4[b] (0.3;8.5)	20.8
Niacin (mg)	15.47	35.0±24.8 (5.1;123.5)	17.3	33.6±22.8 (1.6;135.6)	15.6
Pantothenic acid (mg)	5	9.4±4.3[b] (2.2;19.9)	9.6	7.4±4.4[b] (0.9;40.7)	25.2
Vitamin B_6 (mg)	1.5	3.3±1.8[b] (0.6;8.0)	9.6	2.7±1.5[b] (0.2;8.4)	18.4

Vitamin C (mg)	90	132.3±142.2[b] (1.8;994.8)	39.2	85.1±88.7[b] (0.0;604.1)	64
Vitamin E (mg)	13	18.6±10.7[b] (1.8;55.2)	26.9	13.2±11.4[b] (0.3;91.5)	55.6
Vitamin K (μg)	70	115.5±312.2 (2.2;1957.2)	68	80.4±233.8 (0.0;3095.2)	71.4
Folate (μg)	250	427.0±232.4[b] (167.6;1434.2)	13.5	317.4±213.9[b] (36.8;1958.9)	35.6
Vitamin B_{12} (μg)	4	7.3±3.9 (0.3;17.2)	13.5	6.3±5.5 (0.6;54.4)	32.9
Vitamin D (μg)	15	3.2±2.5 (0.4;11.3)	100	3.0±3.0 (0.0;18.1)	98

[aa]- the same letters for statistically significant difference for the same observed category. between females dietary supplement users and non-users. of the same age; [bb]-the same letters for statistically significant difference for the same observed category. between young men dietary supplement users and non-users. of the same age

Table S4. The average achieved intake of minerals in girls and boys who are dietary supplement users ($n_{girls\ 15/16\ Years}$ = 44. $n_{boys\ 15/16\ Years}$ = 60) and non-users ($n_{girls\ 15/16\ Years}$ = 261. $n_{boys\ 15/16\ Years}$ = 242) at the age of 15/16 years old. Results are presented as mean ± standard deviation (minimum; maximum). Additionally. statistically significant difference (α = 0.05) is indicated. The percentage of respondents (users and non-users of dietary supplements) who did not achieve the recommended daily intake according to USDA recommendations (with a tolerance of 10%) is also shown.

The average achieved intake of minerals in dietary supplement users and non-users at the age of 15/16 years old					
Girls		Dietary supplement users (n = 44)		Dietary supplement non-users (n = 261)	
Minerals	Recommended intake	Achieved intake	% below recommendations	Achieved intake	% below recommendations
Calcium (mg/1000 kcal)	650	526.8±336.9 (153.7;2089.1)	70.4	473.4±245.4 (93.2;2210.0)	75.9
Iron (mg/1000 kcal)	8	4.7±2.2[a] (2.7;16.2)	97.7	5.9±3.9[b] (1.1;29.0)	81.6
Phosphorus (mg/1000 kcal)	620	656.7±257.1 (189.9;1684.5)	36.4	622.5±183.7 (95.5;1411.8)	38.3
Selenium (mg/1000 kcal)	28	32.0±18.2 (9.1;103.2)	45.4	35.2±20.9 (4.7;157.5)	36.4
Zinc (mg/1000 kcal)	6	4.6±1.5 (1.6;8.0)	70.4	4.9±2.6 (1.5;23.7)	72.8
Sodium (mg/1000 kcal)	1200	1311.9±501.6 (233.7;2284.3)	34.1	1360.9±533.6 (377.7;3576.7)	32.6
Boys		Dietary supplement users (n = 60)		Dietary supplement non-users (n = 242)	
Minerals	Recommended intake	Achieved intake	% under recommendations	Achieved intake	% under recommendations
Calcium (mg/1000 kcal)	460	377.8±147.5 (105.8;864.6)	61.7	428.9±173.6 (87.2;1135.9)	51.2
Iron (mg/1000 kcal)	4.5	5.9±4.3 (2.6;35.0)	25	5.8±3.7 (2.6;30.9)	30.2
Phosphorus (mg/1000 kcal)	450	615.8±137.5 (333.6;876.4)	10	650.6±156.3 (28.1;1255.8)	4.5
Selenium (mg/1000 kcal)	20	37.2±18.1 (9.0;108.6)	11.7	36.9±18.4 (1.0;117.4)	14.0
Zinc (mg/1000 kcal)	5	4.8±1.5 (2.2;10.2)	51.7	4.9±1.9 (1.7;18.7)	47.1
Sodium (mg/1000 kcal)	1200	1426.6±524.9 (329.1;3462.3)	26.7	1492.1±582.9 (562.9;4325.4)	26.4

[ab]-different letters for statistically significant difference between girls. dietary supplement users and non-users. of the same age. for the same observed category

Table S5. The average achieved intake of minerals in girls and boys who are dietary supplement users ($n_{girls\ 18/19\ Years}$ = 75. $n_{boys\ 18/19\ Years}$ = 52) and non-users ($n_{girls\ 18/19\ Years}$ = 230. $n_{boys\ 18/19\ Years}$ = 250) at the age of 18/19 years old. Results are presented as mean ± standard deviation (minimum; maximum). The percentage of respondents (users and non-users of food supplements) who did not achieve the recommended daily intake according to USDA recommendations (with a tolerance of 10%) is also shown.

**The average achieved intake of minerals in dietary supplement users and non-users at the age of 18/19 years old**					
Girls		Dietary supplement users (n = 75)		Dietary supplement non-users (n = 230)	
Minerals	Recommended intake	Achieved intake	% below recommendations	Achieved intake	% below recommendations
Calcium (mg/1000 kcal)	650	463.8±202.9 (127.2;1117.5)	73.3	443.2±177.5 (77.3;983.7)	79.6
Iron (mg/1000 kcal)	8	5.6±2.1 (2.7;13.9)	84	5.8±3.2 (1.7;20.5)	81.7
Phosphorus (mg/1000 kcal)	620	658.5±181.3 (334.1;1096.0)	34.7	669.0±203.7 (60.1;1307.0)	27.0
Selenium (mg/1000 kcal)	28	40.4±22.0 (8.7;100.2)	22.7	40.3±23.7 (1.2;142.2)	27.0
Zinc (mg/1000 kcal)	6	5.2±2.0 (2.7;13.5)	62.7	5.3±2.6 (1.1;21.3)	60.9
Sodium (mg/1000 kcal)	1200	1589.9±612.9 (441.2;3466.9)	20	1523.9±740.7 (353.4;4969.3)	31.3
Boys		Dietary supplement users (n = 52)		Dietary supplement non-users (n = 250)	
Minerals	Recommended intake	Achieved intake	% below recommendations	Achieved intake	% below recommendations
Calcium (mg/1000 kcal)	460	425.9±157.6 (136.6;947.4)	48.1	433.5±205.1 (67.3;1186.8)	55.2
Iron (mg/1000 kcal)	4.5	5.7±2.3 (2.6;14.0)	19.2	5.6±2.8 (1.7;23.6)	25.2
Phosphorus (mg/1000 kcal)	450	658.1±183.8a (223.1;1138.6)	3.8	715.7±186.8b (177.4;1294.6)	4.4
Selenium (mg/1000 kcal)	20	41.4±21.1 (10.4;103.7)	11.5	48.3±26.3 (2.8;161.9)	7.2
Zinc (mg/1000 kcal)	5	5.3±1.8 (2.4;11.4)	36.5	5.6±2.4 (1.6;27.8)	33.2
Sodium (mg/1000 kcal)	1200	1786.0±869.3 (434.9;6333.7)	13.5	1601.3±604.7 (237.5;3455.7)	22.8

[ab]-different letters for statistically significant difference between boys. dietary supplement users and non-users. of the same age. for the same observed category

Tables S6. Average achieved mineral intake in girls and boys. dietary supplement users ($n_{girls\ 15/16\ Years}$ = 44. $n_{boys\ 15/16\ Years}$ = 60; $n_{girls\ 18/19\ Years}$ = 75. $n_{boys\ 18/19\ Years}$ = 52) and non-users ($n_{girls\ 15/16\ Years}$ = 261. $n_{boys\ 15/16\ Years}$ = 242; $n_{girls\ 18/19\ Years}$ = 230. $n_{boys\ 18/19\ Years}$ = 250) at both ages. Results are presented as mean±standard deviation (minimum; maximum). Additionally. statistically significant similarity or difference (α = 0.05) is indicated. The percentage of respondents (users and non-users of food supplements) who did not achieve the recommended daily intake according to EFSA recommendations (with a tolerance of 10%) is also shown.

**Average achieved mineral intake in dietary supplement users and non-users at both ages**					
1st measurement (15/16 Years)					
Girls		Dietary supplement users		Dietary supplement non-users	
	Recommended intake	Achieved intake	% under recommendations	Achieved intake	% under recommendations
Calcium (mg)	960	959.7±626.2[a] (218.9;3399.0)	54.5	734.2±457.7[a] (139.1;3939.3)	72.0
Iron (mg)	7	9.2±5.4 (2.7;27.5)	38.6	9.8±8.8 (1.2;79.1)	40
Phosphorus (mg)	640	1261.8±703.0[a] (191.1;4000.6)	13.6	1019.0±564.8[a] (119.0;4426.3)	17.2
Magnesium (mg)	250	260.7±120.5 (89.0;609.7)	54.5	223.6±117.9 (46.1;952.3)	62.1
Selenium (mg)	70	63.2±49.8 (15.0;253.1)	59.1	56.2±36.5 (5.5;196.1)	61.7
Zinc (mg)	9.9	8.7±4.7 (2.5;27.7)	63.6	8.1±5.8 (1.1;44.9)	69.7
Sodium (mg)	2000	2543.2±1536.1 (356.5;8678.5)	34.1	2197.1±1250.0 (141.5;9162.4)	41.0
Boys					
	Recommended intake	Achieved intake	% under recommendations	Achieved intake	% under recommendations
Calcium (mg)	960	1066.8±571.0 (171.6;2294.9)	41.7	1024.3±498.6 (102.8;2938.4)	40.1
Iron (mg)	8	16.9±13.0 (4.8;73.0)	16.7	14.2±10.9 (2.2;96.4)	20.3
Phosphorus (mg)	640	1733.5±813.7 (492.1;3804.1)	5	1611.9±739.6 (34.1;4577.3)	3.3
Magnesium (mg)	300	352.7±157.4 (114.4;812.6)	36.7	315.1±144.9 (53.3;1079.2)	44.2
Selenium (mg)	70	101.5±57.7 (13.5;299.1)	25	91.5±55.3 (1.2;370.1)	35.1
Zinc (mg)	11.8	13.6±7.3 (3.7;37.0)	43.3	12.0±6.3 (2.1;50.3)	49.6
Sodium (mg)	2000	4051.9±2232.0 (551.2;10426.9)	13.3	3669.4±2022.4 (514.9;12511.8)	14.9
2nd measurement (18/19 Years)					
Girls		Dietary supplement users		Dietary supplement non-users	
	Recommended intake	Achieved intake	% under recommendations	Achieved intake	% under recommendations
Calcium (mg)	860	752.6±358.5 (188.6;2541.2)	56	685.7±364.1 (53.8;2541.5)	67
Iron	7	9.6±4.8	28	9.1±5.8	40.4

(mg)		(2.1;20.5)		(0.9;41.7)	
Phosphorus (mg)	550	1110.2±471.8 (281.6;2593.7)	6.7	1048.6±498.5 (69.6;3346.6)	8.3
Magnesium (mg)	300	244.0±105.9 (62.4;576.2)	68	233.8±99.6 (38.9;617.8)	72.6
Selenium (mg)	70	66.9±40.2 (8.5;217.2)	53.3	63.0±40.5 (1.4;194.2)	59.6
Zinc (mg)	8.2	8.8±4.3 (2.0;22.8)	44	8.4±4.9 (1.0;33.5)	52.6
Sodium (mg)	2000	2685.7±1396.1 (400.2;8727.3)	25.3	2372.6±1429.8 (260.5;10219.1)	40
			Boys		
	Recommended intake	Achieved intake	% under recommendations	Achieved intake	% under recommendations
Calcium (mg)	860	1163.7±637.8 (306.9;3881.2)	21.1	1099.4±624.5 (146.7;3534.0)	39.6
Iron (mg)	6	15.0±6.2 (4.5;32.6)	1.9	14.7±9.5 (0.9;65.9)	7.6
Phosphorus (mg)	550	1880.4±943.0 (315.4;4457.9)	1.9	1855.9±862.9 (211.2;4976.6)	1.2
Magnesium (mg)	350	336.4±127.1 (146.4;737.6)	48.1	351.5±175.3 (65.2±1116.4)	48.4
Selenium (mg)	70	119.4±81.7 (16.6;367.9)	26.9	126.2±83.6 (4.2;457.8)	24
Zinc (mg)	10.1	15.0±8.0 (4.2;38.9)	23.1	14.6±8.0 (1.3;64.7)	28
Sodium (mg)	2000	5070.5±4000.2 (1754.9;27783.3)	5.9	4216.1±2359.0 (425.0;13545.2)	10

[aa]- the same letters for statistically significant difference for the same observed category. between females dietary supplement users and non-users. of the same age

Tables S7: Energy and nutritional value of Cedevita instant drink per 100 g and 19 g (recommended daily dose) of powder.

Average nutritional value of Cedevita at		
	100 g	19 g (one serving)
Energy (kcal)	366	65
Carbohydrates (g)	84	16
of which sugars (g)	84	16
Fats (g)	0	0
of which saturated fat (g)	0	0
Proteins (g)	0	0
Sodium (g)	2.3	0.4
Vitamins		
Vitamin C (mg)	213	40
Niacin (mg)	43	8
Vitamin E (mg alfa-TE)	32	6
Pantothenic acid (mg)	16	3
Vitamin B_6 (mg)	3.7	0.7
Riboflavin (mg)	3.7	0.7
Thiamine (mg)	2.9	0.55
Folic acid (µg)	533	100
Vitamin B_{12} (µg)	6.7	1.25

Table S8: Contribution of vitamins from Cedevita to the total intake of vitamins in dietary supplement users (girls and boys) aged 15/16 and 18/19. Results are presented as mean ± standard deviation (minimum; maximum).

**Contribution (%) of vitamins from Cedevita in the total intake of vitamins**				
	1st measurement (15/16 y)		2nd measurement (18/19 y)	
	Girls	Boys	Girls	Boys
Thiamin	29.3±15.9 (7.2;80.0)	31.0±18.9 (7.9;81.7)	32.4±26.1 (12.3;159.9)	56.4±101.6 (9.6;420.3)
Riboflavin	29.5±12.6 (10.7;75.7)	32.8±20.5 (7.2;82.5)	37.5±32.2 (15.0;193.9)	61.4±117.0 (7.9;553.3)
Niacin	58.5±48.7 (9.5;199.5)	68.7±120.6 (6.2;682.8)	55.3±70.1 (8.0;294.6)	108.9±215.9 (5.2;1026.2)
Folic acid	26.0±13.8 (3.6;60.8)	31.6±18.1 (5.6;90.9)	35.0±19.0 (10.9;105.1)	68.7±125.7 (7.4;556.7)
Pantothenic acid	37.2±17.2 (12.4;93.4)	37.6±21.8 (6.2;91.1)	43.2±29.6 (17.9;180.2)	75.4±132.0 (12.3;625.6)
Vitamin B_6	30.4±14.7 (6.4;58.9)	29.8±20.8 (5.7;85.3)	31.2±22.7 (11.1;123.5)	64.5±122.3 (6.9;576.2)
Vitamin B_{12}	24.3±14.1 (6.8;66.8)	28.9±22.2 (6.2;95.7)	33.1±35.3 (8.7;203.4)	48.0±84.4 (6.0;383.0)
Vitamin C	37.0±28.8 (2.3;101.4)	42.0±26.6 (8.8;100.5)	50.9±26.5 (10.2;98.1)	95.2±165.2 (10.7;709.3)
Vitamin E	37.3±18.3 (11.6;95.6)	39.6±21.6 (7.3±89.4)	48.8±39.0 (7.9;232.7)	87.2±151.0 (7.7;590.6)

Table S9. Results obtained by logistic regression taking into account quantitative variables (body mass. body mass index. energy expanditure. sum of thickness of four skin folds. intake of micronutrients. intake of fruits and vegetables. fast food and sweetened beverages) for both genders in both age groups to the binary response of the consumption of vitamin. multivitamin and multivitamin-mineral preparations.

Source	DF	Chi-square (Wald)	Pr > Wald	Chi-square (LR)	Pr > LR	Odds ratio
15/16 years – Vitamin and multivitamin preparations						
Body mass (kg)	1	4.289	0.038	4.321	0.038	1.050
Body mass index (kg/m^2)	1	7.588	0.006	7.879	0.005	0.756
Sum of thickness of 4 skin folds (mm)	1	2.034	0.154	2.000	0.157	1.020
Energy expanditure (kcal/kg/dan)	1	0.063	0.801	0.000	1.000	1.017
Thiamine (mg/1000 kcal)	1	2.579	0.108	2.528	0.112	2.103
Riboflavin (mg/1000 kcal)	1	0.310	0.578	0.316	0.574	0.808
Niacin (mg/1000 kcal)	1	17.422	<0.0001	19.418	<0.0001	0.879
Pantothenic acid (mg/1000 kcal)	1	10.920	0.001	12.621	0.000	1.469
Vitamin B_6 (mg/1000 kcal)	1	5.992	0.014	5.556	0.018	2.394
Vitamin C (mg/1000 kcal)	1	0.262	0.609	0.258	0.611	1.001
Vitamin E (mg/1000 kcal)	1	0.050	0.824	0.049	0.824	1.008
Vitamin K (µg/1000 kcal)	1	0.340	0.560	0.398	0.528	0.998
Folate (µg/1000 kcal)	1	19.609	<0.0001	19.963	<0.0001	1.005
Vitamin B_{12} (µg/1000 kcal)	1	1.740	0.187	1.689	0.194	1.102
Vitamin D (µg/1000 kcal)	1	0.237	0.626	0.243	0.622	0.930
Calcium (mg/1000 kcal)	1	0.052	0.820	0.052	0.820	1.000
Iron (mg/1000 kcal)	1	0.522	0.470	0.574	0.449	0.972
Magnesium (mg/1000 kcal)	1	0.125	0.724	0.122	0.726	1.002
Phosphorus (mg/1000 kcal)	1	0.124	0.725	0.125	0.724	1.000
Potassium (mg/1000 kcal)	1	0.011	0.917	0.011	0.917	1.000
Selenium (mg/1000 kcal)	1	0.194	0.659	0.197	0.657	0.996
Zinc (mg/1000 kcal)	1	0.718	0.397	0.777	0.378	0.931
Sodium (mg/1000 kcal)	1	0.700	0.403	0.718	0.397	1.000
Fruits and vegetables (g/1000 kcal)	1	5.195	0.023	5.828	0.016	0.131
Fast food (g/1000 kcal)	1	0.261	0.609	0.266	0.606	0.542
Non-carbonated sweetened drinks (g/1000 kcal)	1	10.599	0.001	10.318	0.001	6.918
Carbonated sweetened drinks (g/1000 kcal)	1	0.441	0.507	0.491	0.484	0.455
15/16 years – Multivitamin-mineral preparations						
Body mass (kg)	1	0.574	0.449	0.581	0.446	0.964
Body mass indeks (kg/m^2)	1	0.519	0.471	0.524	0.469	0.859
Sum of thickness of 4 skin folds (mm)	1	0.007	0.935	0.007	0.935	1.003
Energy expenditure (kcal/kg/dan)	1	0.042	0.837	0.000	1.000	0.974
Thiamine (mg/1000 kcal)	1	3.053	0.081	3.295	0.069	0.153
Riboflavina (mg/1000 kcal)	1	5.660	0.017	4.982	0.026	4.501
Niacin (mg/1000 kcal)	1	2.581	0.108	2.630	0.105	0.910
Pantothetic acid (mg/1000 kcal)	1	1.328	0.249	1.390	0.238	1.283
Vitamin B_6 (mg/1000 kcal)	1	0.044	0.834	0.042	0.838	1.200
Vitamin C (mg/1000 kcal)	1	0.297	0.586	0.333	0.564	0.997
Vitamin E (mg/1000 kcal)	1	0.344	0.557	0.361	0.548	0.962

Vitamin K (μg/1000 kcal)	1	0.062	0.804	0.055	0.815	1.001
Folate (μg/1000 kcal)	1	0.077	0.781	0.080	0.777	0.999
Vitamin B_{12} (μg/1000 kcal)	1	3.402	0.065	2.808	0.094	1.221
Vitamin D (μg/1000 kcal)	1	0.000	0.991	0.000	0.991	1.002
Calcium (mg/1000 kcal)	1	3.608	0.057	3.623	0.057	1.003
Iron (mg/1000 kcal)	1	0.088	0.766	0.096	0.756	0.978
Magnesium (mg/1000 kcal)	1	1.985	0.159	2.747	0.097	0.983
Phosphorus (mg/1000 kcal)	1	0.165	0.685	0.169	0.681	1.001
Potassium (mg/1000 kcal)	1	0.179	0.672	0.179	0.672	1.000
Selenium (mg/1000 kcal)	1	0.536	0.464	0.574	0.449	0.987
Zinc (mg/1000 kcal)	1	0.731	0.393	0.664	0.415	1.113
Sodium (mg/1000 kcal)	1	0.002	0.965	0.002	0.965	1.000
Fruits and vegetables (g/1000 kcal)	1	0.183	0.669	0.192	0.662	0.489
Fast food (g/1000 kcal)	1	2.079	0.149	1.812	0.178	13.335
Non-carbonated sweetened drinks (g/1000 kcal)	1	0.043	0.835	0.042	0.838	1.276
Carbonated sweetened drinks (g/1000 kcal)	1	0.012	0.912	0.013	0.911	0.817
18/19 years – Vitamin and multivitamin preparations						
Body mass (kg)	1	0.066	0.797	0.066	0.797	0.995
Body mass indeks (kg/m²)	1	0.011	0.918	0.011	0.918	0.992
Sum of thickness of 4 skin folds (mm)	1	0.073	0.786	0.074	0.786	0.997
Energy expenditure (kcal/kg/dan)	1	0.042	0.837	0.000	1.000	0.986
Thiamine (mg/1000 kcal)	1	0.293	0.588	0.293	0.588	1.239
Riboflavin (mg/1000 kcal)	1	0.006	0.936	0.006	0.936	1.032
Niacin (mg/1000 kcal)	1	6.012	0.014	6.394	0.011	0.938
Pantothetic acid (mg/1000 kcal)	1	5.003	0.025	5.339	0.021	1.290
Vitamin B_6 (mg/1000 kcal)	1	3.468	0.063	3.459	0.063	2.160
Vitamin C (mg/1000 kcal)	1	0.061	0.805	0.062	0.803	0.999
Vitamin E (mg/1000 kcal)	1	11.695	0.001	11.646	0.001	1.119
Vitamin K (μg/1000 kcal)	1	0.884	0.347	1.070	0.301	0.999
Folate (μg/1000 kcal)	1	10.198	0.001	9.966	0.002	1.004
Vitamin B_{12} (μg/1000 kcal)	1	1.254	0.263	1.263	0.261	<0.01
Vitamin D (μg/1000 kcal)	1	0.560	0.454	0.548	0.459	1.093
Calcium (mg/1000 kcal)	1	0.207	0.649	0.206	0.650	1.000
Iron (mg/1000 kcal)	1	0.066	0.797	0.068	0.795	0.989
Magnesium (mg/1000 kcal)	1	0.474	0.491	0.475	0.491	0.997
Phosphorus (mg/1000 kcal)	1	1.316	0.251	1.314	0.252	0.999
Potassium (mg/1000 kcal)	1	0.028	0.868	0.028	0.868	1.000
Selenium (mg/1000 kcal)	1	0.001	0.975	0.001	0.975	1.000
Zinc (mg/1000 kcal)	1	0.027	0.870	0.027	0.870	0.990
Sodium (mg/1000 kcal)	1	1.589	0.207	1.549	0.213	1.000
Fruits and vegetables (g/1000 kcal)	1	1.132	0.287	1.179	0.277	0.417
Fast food (g/1000 kcal)	1	0.345	0.557	0.336	0.562	1.897
Non-carbonated sweetened drinks (g/1000 kcal)	1	15.086	0.000	15.713	<0.0001	39.175
Carbonated sweetened drinks (g/1000 kcal)	1	2.889	0.089	3.113	0.078	0.122
18/19 years – Multivitamin-mineral preparations						
Body mass (kg)	1	1.881	0.170	1.878	0.171	1.058

Body mass indeks (kg/m^2)	1	0.447	0.504	0.458	0.498	0.898
Sum of thickness of 4 skin folds (mm)	1	0.000	0.988	0.000	0.988	1.000
Energy expenditure (kcal/kg/dan)	1	3.550	0.060	0.000	1.000	1.201
Thiamine (mg/1000 kcal)	1	0.027	0.869	0.028	0.868	0.904
Riboflavin (mg/1000 kcal)	1	0.000	0.984	0.000	0.984	1.016
Niacin (mg/1000 kcal)	1	0.944	0.331	0.964	0.326	0.959
Pantothetic acid (mg/1000 kcal)	1	0.110	0.740	0.110	0.740	1.069
Vitamin B_6 (mg/1000 kcal)	1	2.504	0.114	2.269	0.132	2.778
Vitamin C (mg/1000 kcal)	1	0.122	0.727	0.115	0.735	1.002
Vitamin E (mg/1000 kcal)	1	0.714	0.398	0.698	0.403	1.057
Vitamin K (µg/1000 kcal)	1	0.338	0.561	0.298	0.585	1.001
Folate (µg/1000 kcal)	1	2.458	0.117	2.235	0.135	1.003
Vitamin B_{12} (µg/1000 kcal)	1	0.002	0.964	0.002	0.964	0.017
Vitamin D (µg/1000 kcal)	1	0.049	0.825	0.051	0.822	0.946
Calcium (mg/1000 kcal)	1	0.637	0.425	0.619	0.431	1.001
Iron (mg/1000 kcal)	1	0.303	0.582	0.343	0.558	0.942
Magnesium (mg/1000 kcal)	1	0.005	0.941	0.005	0.941	1.001
Phosphorus (mg/1000 kcal)	1	1.044	0.307	1.034	0.309	0.998
Potassium (mg/1000 kcal)	1	0.334	0.563	0.328	0.567	1.000
Selenium (mg/1000 kcal)	1	0.055	0.815	0.054	0.816	1.003
Zinc (mg/1000 kcal)	1	0.597	0.440	0.541	0.462	1.091
Sodium (mg/1000 kcal)	1	1.795	0.180	1.647	0.199	1.000
Fruits and vegetables (g/1000 kcal)	1	0.185	0.667	0.195	0.659	0.470
Fast food (g/1000 kcal)	1	0.027	0.869	0.028	0.868	0.678
Non-carbonated sweetened drinks (g/1000 kcal)	1	0.009	0.923	0.009	0.924	1.231
Carbonated sweetened drinks (g/1000 kcal)	1	0.365	0.546	0.423	0.516	0.153
